# Inhibition of GSK3β is synthetic lethal with FHIT loss in lung cancer by blocking homologous recombination repair

**DOI:** 10.1038/s12276-024-01374-0

**Published:** 2025-01-06

**Authors:** Shishi Tao, Yue Pu, Eun Ju Yang, Guowen Ren, Changxiang Shi, Li-Jie Chen, Liang Chen, Joong Sup Shim

**Affiliations:** 1 Cancer Centre, Faculty of Health Sciences, University of Macau, Taipa, Macau SAR China; 2 Institute of Cancer Research, Shenzhen Bay Laboratory, Shenzhen, 518055 Guangdong China; 3 Nanjing Key Laboratory of Female Fertility Preservation and Restoration, Nanjing Women and Children’s Healthcare Institute, Women’s Hospital of Nanjing Medical University (Nanjing Women and Children’s Healthcare Hospital), Nanjing, 210004 China; 4 Shenzhen Laboratory of Tumor Cell Biology, Institute of Biomedicine and Biotechnology, Shenzhen Institute of Advanced Technology, Chinese Academy of Sciences, Shenzhen, 518055 China; 5 MOE Frontiers Science Centre for Precision Oncology, University of Macau, Taipa, Macau SAR China

**Keywords:** Targeted therapies, Oncogenes

## Abstract

FHIT is a fragile site tumor suppressor that is primarily inactivated upon tobacco smoking. FHIT loss is frequently observed in lung cancer, making it an important biomarker for the development of targeted therapy for lung cancer. Here, we report that inhibitors of glycogen synthase kinase 3 beta (GSK3β) and the homologous recombination DNA repair (HRR) pathway are synthetic lethal with FHIT loss in lung cancer. Pharmacological inhibition or siRNA depletion of GSK3β selectively suppressed the growth of FHIT-deficient lung cancer tumors in vitro and in animal models. We further showed that FHIT inactivation leads to the activation of DNA damage repair pathways, including the HRR and NHEJ pathways, in lung cancer cells. Conversely, FHIT-deficient cells are highly dependent on HRR for survival under DNA damage stress. The inhibition of GSK3β in FHIT-deficient cells suppressed the ATR/BRCA1/RAD51 axis in HRR signaling via two distinct pathways and suppressed DNA double-strand break repair, leading to the accumulation of DNA damage and apoptosis. Small molecule inhibitors of HRR, but not NHEJ or PARP, induced synthetic lethality in FHIT-deficient lung cancer cells. The findings of this study suggest that the GSK3β and HRR pathways are potential drug targets in lung cancer patients with FHIT loss.

## FHIT Loss Reveals Vulnerabilities in Lung Cancer Therapy

Lung cancer is a major cause of cancer deaths, often due to smoking. Despite progress, death rates remain high. Researchers found a gap in targeting FHIT, a tumor suppressor gene often missing in lung cancer. The study used synthetic lethality (a genetic interaction where two gene mutations cause cell death) to target FHIT loss. They tested drugs on lung cancer cells and discovered that blocking GSK3β (an enzyme involved in various cell processes) killed FHIT-deficient cells. This study used cell cultures and mice, focusing on DNA repair pathways vital for cancer cell survival. Results showed that inhibiting GSK3β increased DNA damage and cell death in FHIT-deficient cells. The study suggests that targeting GSK3β could lead to new treatments for lung cancer with FHIT loss, offering hope for better therapies.

This summary was initially drafted using artificial intelligence, then revised and fact-checked by the author.

## Introduction

Lung cancer is one of the most common primary malignancies and the leading cause of cancer-related death worldwide^1^. Although recent advances in targeted therapy and immunotherapy have improved patient survival and quality of life, lung cancer still has the highest mortality rate among all types of cancer.

The number one risk factor for lung cancer is tobacco smoking. Over 90% of lung cancer cases among men and over 80% among women worldwide are attributable to tobacco smoking^2^. Tobacco smoke is known to release at least 70 known chemical carcinogens, such as N-nitrosamines, aromatic amines and benzene, that can cause DNA damage and gene mutations. Tobacco smoke primarily damages one of the fragile sites in the genome, FRA3B, which is located at chromosome 3p14.2^3^. Molecular characterization of the FRA3B fragile site revealed the presence of the tumor suppressor FHIT (Fragile Histidine Triad) at this locus. FHIT is highly susceptible to deletion upon exposure to genotoxic stress, such as tobacco smoke^4^. Loss of FHIT is frequently observed in most cancers, particularly in lung cancer, accounting for >80% of lung cancer cases^5^. Moreover, the loss of FHIT is significantly greater in the tumors of smokers (75%) than in those of nonsmokers (39%)^6^, suggesting that FHIT loss is a key event during smoke-induced lung cancer carcinogenesis and development.

Heterozygote or homozygous deletion of Fhit in mice results in increased susceptibility to tumor development^7^, whereas FHIT gene delivery into Fhit+/− mice significantly inhibits tumor development induced by chemical carcinogens^8^. FHIT loss is associated with decreased apoptosis^9^, increased EMT and metastatic potential, and chemotherapy resistance^10–12^. Restoration of FHIT expression induced apoptosis and suppressed tumorigenicity in lung, cervical and breast cancer models^13,14^, suggesting that FHIT is a bona fide tumor suppressor.

Although the molecular functions of FHIT in cancer have been investigated for decades, the mechanisms of its tumor suppressive effects remain elusive. FHIT has diadenosine triphosphate hydrolase (Ap3Aase) activity and is thought to be involved in nucleotide metabolism and RNA processing^15^. Several proteins that interact with FHIT in cancer cells have also been identified, including heat shock protein (HSP) family proteins^16^, Annexin A family (ANXA) proteins^17^ and β-catenin^18^, which supports the potential role of FHIT in cancer signaling. On the other hand, FHIT, as a sensor of fragile site DNA damage, functions as a genome caretaker whose loss leads to increased replication stress, genome instability and accumulation of genetic alterations^19–21^. In line with these observations, FHIT loss was shown to modulate ATR/CHK1 signaling and the DNA damage response^22,23^. These observations suggest that FHIT is a tumor suppressor that controls smoke-induced lung carcinogenesis and lung cancer metastasis and could be an important biomarker for the development of targeted lung cancer therapy. However, no actual targets or drugs targeting FHIT loss have been developed, despite the long-term investigation of FHIT in lung cancer.

In an attempt to exploit FHIT loss as a biomarker for the discovery of lung cancer targets, we conducted a synthetic lethal drug screen in FHIT-engineered lung cancer cell lines. In this study, we found that GSK3β inhibition is synthetic lethal with FHIT loss in lung cancer cells. Further mechanistic experiments revealed the homologous recombination DNA repair (HRR) pathway as the crucial downstream target of GSK3β in the synthetic lethality. This study highlights that GSK3β, as well as the HRR pathway, is a potential actionable target in FHIT-deficient lung cancer and provides the first candidate for FHIT-targeting synthetic lethal therapy for smokers with lung cancer.

## Materials and methods

### Cell culture

Two FHIT-expressing (HCC827 and H1650) and two FHIT-defective (H1299 and H460) lung cancer cell lines were purchased from the American Type Culture Collection (ATCC, USA). RPMI 1640 medium containing 1% penicillin/streptomycin (P/S) and 10% fetal bovine serum (FBS) was used to culture these cells at 37 °C with 5% CO_2_. All of the cell culture media and supplements were obtained from Life Technologies (USA).

### Chemicals and plasmids

The GSK3β inhibitor CHIR99021 (DC1023), the ATR inhibitor VE-821 (DC3132), and the DNA-PK inhibitor NU-7441 (DC3100) were purchased from DC Chemical (Shanghai, China); the ATR inhibitor elimusertib (S9864) was purchased from MedChemExpress (Shanghai, China); and cycloheximide (CHX) was obtained from Sigma‒Aldrich (USA). The Fhit-HDR plasmid (sc-404220-HDR) and Fhit CRISPR/Cas9 KO plasmid (sc-404220) were purchased from Santa Cruz. pCDH-EF1-FHC was a gift from Richard Wood (Addgene plasmid # 64874)^24^, pLCN DSB Repair Reporter (DRR) (Addgene plasmid # 98895) and pCAGGS DRR mCherry Donor EF1a BFP (Addgene plasmid # 98896) were gifts from Jan Karlseder^25^, and pCBASceI was a gift from Maria Jasin (Addgene plasmid # 26477)^26^. The lentiviral packaging plasmids pCMV-VSV-G (Addgene plasmid # 8454) and pCMV-dR8.2 dvpr (Addgene plasmid # 8455) were gifts from Bob Weinberg^27^. FHIT WT (CS-R0029-M14) and FHIT mutant H96N (CS-R0029-M14-01) plasmids were purchased from GeneCopoeia (Guangzhou, China).

### Antibodies

Antibodies against the following proteins were used: FHIT (JP18163, IBL International), HA (3724 s, CST), GSK3α (AG2065, Beyotime), GSK3β (9832 s, CST), pho-GSK3α/β (Tyr279/216) (AF1522, Beyotime), PARP (9532 s, CST), cleaved-caspase3 (9664 s, CST), γ-H2A. X (9718 s, CST), RAD51 (PA5-27195, Invitrogen), BRCA1 (sc-6954, Santa Cruz), p-BRCA1 (s1423) (AB600, Beyotime), HSP90 (37-9400, Thermo Fisher), DNA-PKCS (sc-5282, Santa Cruz), pho-DNA-PK (Thr2609) (PA1-29541, Invitrogen), ATR (13934 s, CST). pho-ATR (s428) (2853 s, CST), Snail (3879, CST), Slug (9585, CST), RELB (4922 s, CST), GAPDH (sc-3650620, Santa Cruz), goat anti-mouse IgG (H + L) peroxidase conjugated (31430, Thermo Scientific), goat anti-rabbit IgG (H + L) peroxidase conjugated (31460, Thermo Scientific), and Alexa Fluor 488 donkey anti-rabbit antibody (A21202, Thermo Fisher). Details of the use of antibodies can be found in Supplementary Table 1.

### Construction of FHIT-knockout cell lines

HCC827 and H1650 cells were used to construct FHIT knockout cell pairs and they were seeded in 12-well plates at a density of 70%. After growth overnight, 0.5 μg of FHIT-HDR plasmid and 0.5 μg of FHIT CRISPR/Cas9 KO plasmid were transfected into each well with Lipo3000 (Thermo Fisher Scientific) according to the manufacturer’s instructions. The medium was replaced after 4–6 h, and green fluorescent protein (GFP) and red fluorescent protein (RFP) were observed under an EVOS fluorescence microscope (Life Technologies) at 24 h after transfection to judge the transfection efficiency, where the CRISPR/Cas9 KO plasmid expressed GFP and the FHIT-HDR plasmid expressed RFP. For another 24–48 h, medium containing 4 μg/mL puromycin was used to culture the cells for 3–5 days to exclude those that failed to be transfected, and the surviving cells were transferred into 96-well plates (1 cell per well). After ten days, single-cell-formed RFP-expressing clusters were transferred, and growth was continued in 12-well plates. Finally, PCR and Western blotting were used to confirm FHIT knockout. The PCR primer sequences are listed in Supplementary Table 2.

### Construction of FHIT-overexpressing cell lines

FHIT cDNA was inserted into the lentiviral plasmid pCDH-EF1-FHC to generate the FHIT-overexpressing plasmid, and HEK-293T cells were grown in a 10 cm dish at a density of 70% to prepare FHIT-overexpressing lentiviruses. Then, FHIT-overexpressing, pCMV-dR8.2 dvpr, and pCMV-VSV-G plasmids were transfected into HEK-293T cells at a ratio of 9 μg:10 μg:1 μg with Lipo3000. Sixteen to 18 h after transfection, the cells were cultured in medium containing 30% FBS for an additional 48 h. The culture medium was collected in a new tube, centrifuged at 1500 rpm for 3 min, the supernatant was collected, and the mixture was passed through 0.45 μM filters to obtain lentiviral particles. The H1299 and H460 lung cancer cell lines were seeded in a 6-well plate at a density of 60%. After attaching overnight, 1 mL of lentiviral particles, 1 mL of medium, and 8 μg/mL polybrene were added. Forty-eight hours after lentiviral infection, the cells were incubated with 4 µg/mL puromycin-containing medium for 3–5 days. Finally, the FHIT-overexpressing clones were verified by qPCR and Western blotting.

### Highly selective inhibitor library screening (318 compounds)

The highly selective inhibitor library was purchased from Selleck Chemicals Company (Houston, TX), and all of the compounds were diluted 3-fold starting from 100 μM to prepare a total of 8 concentrations, which were added to 384-well plates (6 μL/well). HCC827 *FHIT*^*+/+*^ and HCC827 *FHIT*^−/−^ cells were seeded into highly selective inhibitor library-containing 384-well plates at 3000 cells/well and incubated for 72 h to test cell viability via the Alamar blue assay as previously described. The IC_50_ value of each compound for FHIT-isogenic cells was calculated with GraphPad Prism 8.0 software. The selectivity index (SI) was determined using the formula SI = IC_50_(*FHIT*^*+/+*^)/IC_50_(*FHIT*^−/−^). Compounds with an SI > 2 were selected for further verification.

### Apoptosis and cell cycle analysis

An Alexa Fluor 488 Annexin V/Dead Cell Apoptosis Kit (Invitrogen) was used to analyze apoptosis. A total of 0.5 × 10^6^ cells were harvested and resuspended in 100 μL of 1 × binding buffer, after which 5 μL of Annexin V and 5 μL of 100 μg/mL propidium iodide (PI) were added to stain the cells for 15 min at RT. For the cell cycle analysis, the cells were collected and then fixed with pre-chilled 70% ethanol for 2 h on ice. After being washed with PBS twice, the cells were incubated with PBS diluted with 100 μg/mL RNase and 5 μg/mL PI for 30 min at RT. A CytoFLEX flow cytometer (Beckman Coulter, USA) was used to analyze the cell samples.

### siRNA transfection

Specific siRNAs against FHIT (hs.Ri.FHIT.13.1), GSK3α (hs.Ri.GSK3A.13.1) and GSK3β (hs.Ri.GSK3B.13.1) were synthesized by Integrated DNA Technologies, diluted to 20 μM with DNAse/RNAse-free UltraPure Distilled Water (Invitrogen, USA), and then transfected into cells with Lipofectamine RNAiMAX reagent (Thermo Fisher Scientific). Appropriate amounts of siRNA and transfection reagents were diluted with Opti-MEM medium separately, followed by mixing and incubation at RT for 10–15 min. The mixture was transferred to a 6-well plate and cocultured with cells at 37 °C in a 5% CO_2_ incubator for 48 h. The siRNA sequences are listed in Supplementary Table 3.

### Reverse transcription and quantitative real-time PCR (RT‒qPCR)

The cells cultured in a 12-well plate were washed with PBS twice and incubated with 150 μL of whole-cell total RNA extraction buffer (10 mM Tris-HCl, pH 7.4; 0.25% Igepal CA-630; 150 mM NaCl) at RT for 10 min^28^. Three microlitres of total RNA was taken from each well for reverse transcription via a High-Capacity cDNA Reverse Transcription Kit (Thermo Fisher Scientific), after which 1 μL of product from each reverse transcription sample was used to perform qPCR with iTaq Universal SYBR Green Supermix (Bio-Rad) reagent in a CFX96 Real-Time PCR System (Bio-Rad, Hercules, CA). The primer sequences are listed in Supplementary Table 4.

### Western blotting

The cells grown in a 6-well plate were washed with PBS twice and lysed for whole-cell protein extraction with 2 × Laemmle lysis buffer (62.5 mM Tris-HCl, pH 6.8; 1% SDS; 10% glycerol; 10% 2-mercaptoethanol; 0.005% bromophenol blue). Tumor tissues from the mouse xenograft model were cut into pieces and homogenized with a Polytron® PT 1200 E Manual Disperser (Kinematica, Bohemia, NY). Then, the tumor tissue was lysed with RIPA buffer (50 mM Tris-HCl, pH 8.0; 0.1% SDS; 150 mM NaCl; 1% Triton X-100; 0.5% sodium deoxycholate; and 1 × protease inhibitor cocktail), and the protein concentration was determined with a BCA protein assay kit (Thermo Fisher Scientific). The following steps were used: 1) Heat the protein samples at 95 °C for 10 min. 2) Separate the protein samples via SDS‒PAGE. 3) Transfer the separated proteins onto a PVDF membrane. 4) Block with 5% fat-free milk at RT for 30 min and wash with PBST 3 times. 5) Incubate with primary antibodies at 4 °C overnight and wash with PBST 3 times. 6) Incubate with HRP-linked secondary antibodies at RT for one hour and wash with PBST 3 times. 7) Capture the labeled protein bands using a ChemiDoc MP imaging system (Bio-Rad, Hercules, CA).

### Immunofluorescence

An equal volume of 4% paraformaldehyde (PFA) was added to 8-well chamber coverslips (Thermo Fisher Scientific) containing growing cells, which were fixed at 37 °C for 30 min. The cells were subsequently permeabilized and blocked with 3% BSA supplemented with 0.1% Triton X-100 at RT for 30 min. Then, the cells were incubated with primary antibody at RT for 2 h, followed by incubation with an Alexa Fluor 488-conjugated secondary antibody at RT for 1 h. After each step, the cells were dipped into PBS three times to remove the residue from the previous step. Fluoromount-GTM containing DAPI (Thermo Fisher Scientific) was used to mount the cells and stain the nuclei for 5 min. Finally, immunofluorescence images were acquired immediately via a Zeiss LSM 710 confocal microscope (Carl Zeiss, Thornwood, NY), or the prepared slides were stored at 4 °C in the dark for a short time (no more than two weeks) before images were acquired.

### Comet Assay

A comet assay was performed on cells treated with a GSK3β inhibitor. The cells cultured in 12-well plates were digested, washed twice with PBS at 800 rpm for 3 min and resuspended in PBS at 1 × 10^4^ cells/mL. A total of 10 μL of the cell suspension and 120 μL of 0.5% low melting point agarose were mixed at 37 °C, after which the mixture was dropped onto a 1.5% agarose-coated slide, covered with a coverslip and solidified for 5 min at 4 °C. The coverslip was removed, and the mixture was lysed with precooled, fresh lysis buffer (2.5 M NaCl, 100 mM EDTA, 10 mM Tris, and 1% Triton X-100, pH=10) in the dark at 4 °C overnight. The slide was transferred to a tank and incubated with precooled, fresh electrophoresis buffer (300 mM NaOH, 1 mM EDTA, pH=10) for 20 min. The slides were then processed for electrophoresis at 300 mA for 30 min on ice. After neutralization with 0.4 M Tris-HCl (pH 7.5) three times, the nuclei were stained with 10 μg/mL PI for 10 min at RT, and images were acquired via an EVOS fluorescence microscope (Life Technologies).

### NHEJ and HRR efficiency assays

DNA double-strand break (DSB) repair reporter (DRR) cells were generated via lentiviral transduction of the pLCN DSB Repair Reporter (DRR), pCMV-dR8.2 dvpr, and pCMV-VSV-G plasmids as described earlier. Lung cancer cells were transduced with lentivirus for 48 h, and stable DRR reporter-expressing cells were selected with 10 µg/mL G418 for 3‒5 days. Successful DRR-expressing cells were verified via PCR detection of the neomycin resistance gene. In accordance with the principles of the HRR and NHEJ efficiency assays, the experiment was performed in DRR cells transiently transfected with 1 μg pCBASceI and 1 μg pCAGGS DRR mCherry Donor EF1a BFP plasmids in 6-well plates. After 24 h, the transfected cells were incubated with compounds for the designated time and collected to analyze the ratio of EGFP (NHEJ efficiency) and mCherry (HRR efficiency) via a CytoFLEX Flow Cytometer (Beckman Coulter, USA). The fluorescence images of the cells were captured with a Nikon A1R confocal microscope (Nikon Corporation, Japan).

### Biotin proximity labeling (Bio-ID) and immunoprecipitation

Cells in 6 cm dishes were transfected with 4 μg of BirA biotin ligase plasmid (Addgene # 36047) and 4 μg of FHIT-BirA plasmid (the FHIT gene was cloned and inserted into the BirA plasmid) in RPMI 1640 medium supplemented with biotin. After 24 h, the cells were washed with PBS twice and lysed with 750 μL of Bio-ID buffer (50 mM Tris-HCl pH 7.4, 500 mM NaCl, 0.4% SDS, 5 mM EDTA, 1 mM DTT, 2% Triton X-100, and 1 × protease inhibitor) on ice for 45 min, followed by ultrasonication. The cell lysates were centrifuged at 12,500 rpm at 4 °C for 10 min, and 75 μL of the supernatant was taken as the input. The remaining supernatant was diluted 2.5-fold with precooled 50 mM Tris-HCL (pH 7.4) and incubated with preequilibrated (Bio-ID lysis buffer:50 mM Tris-HCL = 1:1) streptavidin beads (Invitrogen, 65002) overnight at 4 °C. The beads were washed with buffer 1 (2% SDS, once), buffer 2 (0.1% deoxycholate, 1% Triton X-100, 50 mM NaCl, 1 mM EDTA, 50 mM HEPES pH 7.5, once), buffer 3 (250 mM LiCl, 0.5% NP40, 0.5% deoxycholate, 1 mM EDTA, 10 mM Tris-HCL pH 8.1, once) and buffer 4 (50 mM NaCl, 50 mM Tris-HCL pH 7.4, twice). SDS‒PAGE sample loading buffer was added to the input and beads, the mixture was heated at 95 °C for 10 min, and the samples were analyzed via Western blotting of FHIT and HSP90 using the indicated antibodies. For immunoprecipitation (IP), HCC827 *FHIT*^−/−^ cells in 6-cm dishes were transfected with or without FHIT overexpression plasmids (GeneCopoeiaCS-R0029-M14). After 24 h, the cells were washed twice with PBS and lysed with 750 μL of IP buffer (Thermo, #87787 and 1 × protease inhibitor) on ice for 45 min, followed by ultrasonication. After centrifugation at 12,500 rpm and 4 °C for 10 min, 75 μL of the supernatant was taken as the input. The remaining supernatants were diluted 2.5-fold with precooled IP buffer and then incubated with 15 μL of preequilibrated protein A/G beads (Thermo, 88802) and 5 μL of mouse IgG (Santacruz, sc-2025) or 5 μL of anti-HSP90 antibody (Thermo, 37–9400) overnight at 4 °C. The beads were washed 5 times with IP buffer, and the IP samples were subjected to Western blotting for FHIT, HSP90, BRCA1 and RAD51.

### Mouse experiments

We kept twenty-four athymic nude mice (female, 7-week, eight mice/group) and generated tumor xenograft mouse models in a specific pathogen-free room in the Animal Facility at the University of Macau. Viable FHIT-isogenic cell pairs were resuspended in matrix gel, and subcutaneous inoculation was performed in the right (3 × 10^6^ HCC827 *FHIT*^*+/+*^ cells/mouse) and left (6 × 10^6^ HCC827 *FHIT*^−/−^ cells/mouse) armpits of the mice. Four days after injection, we observed that the implanted tumor had grown to approximately 100 mm^3^ and treated the mice with daily intraperitoneal injections of vehicle (sterile saline containing 5% dimethyl sulfoxide, 5% Tween 80, or 5% polyethylene glycol-400) or vehicle containing CHIR99021 (30 or 60 mg/kg) for 21 days. During this period, we measured the body weight and long and short axes of the tumors every three days to assess drug toxicity and drug efficacy. We also closely monitored mouse activity. The tumor volume was calculated via a modified ellipsoid formula (long axis × short axis^2^/2). At the end of the drug treatment, tumor tissues were obtained from euthanized mice for weighing and further analysis. The Animal Research Ethics Committee of the University of Macau approved the animal experiments, and we followed the ARRIVE Guidelines^29^.

### Data analysis

All of the experiments were performed in at least three replicates, and similar results were observed. Statistical differences in the tumor volume between the two groups were analyzed via two-way ANOVA, and other statistically significant differences between the two groups were determined via the unpaired two-tailed Student’s t test with GraphPad Prism 8.0 software.

## Results

### A screen of a highly selective inhibitor compound library identified GSK3β as a synthetic lethal target for FHIT loss in lung cancer cells

**Fig. 1:**
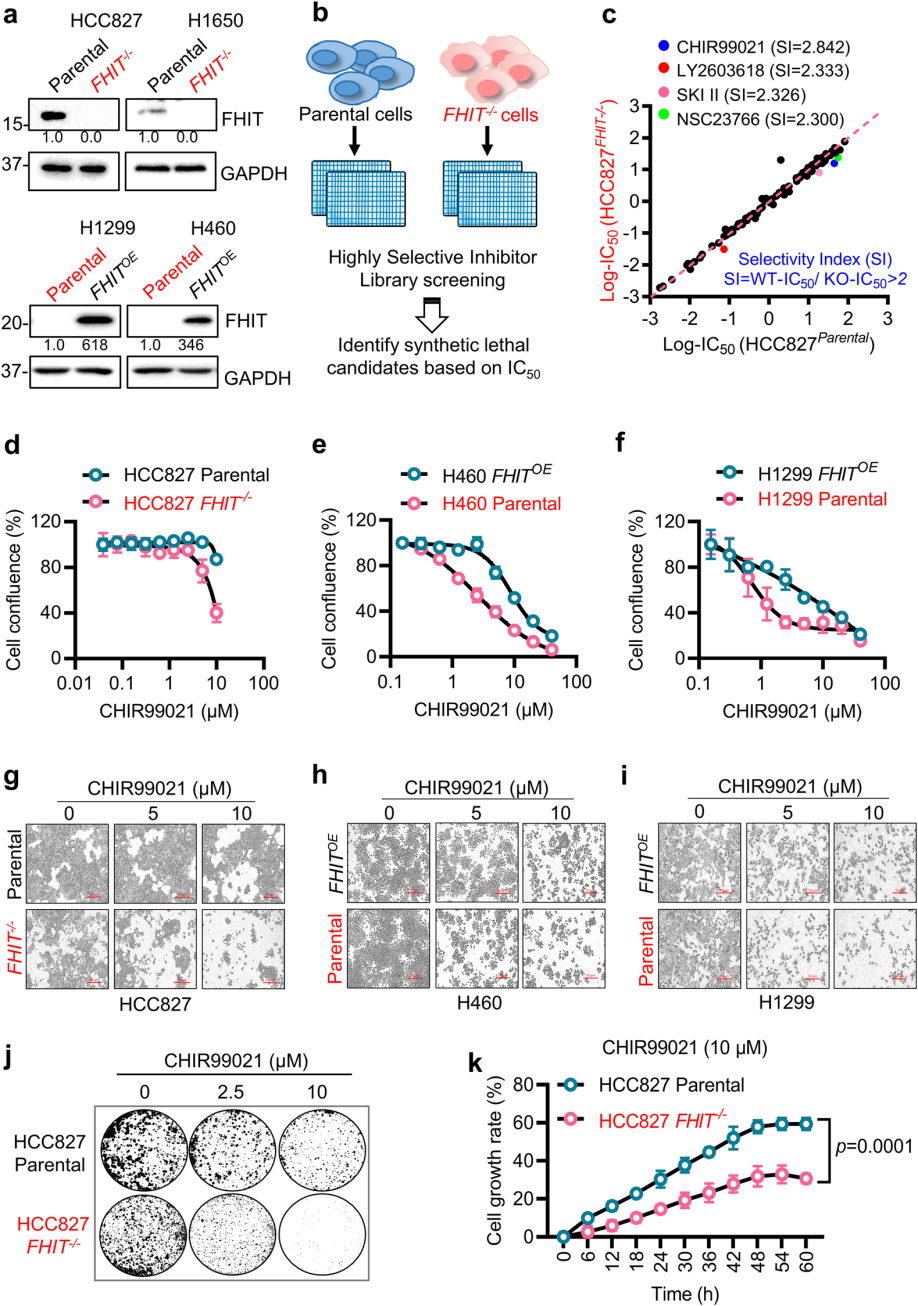
Discovery of synthetic lethality between GSK3β and FHIT through highly selective inhibitor library screening. **a** Western blotting was used to verify FHIT knockout and overexpression in lung cancer cell pairs. HCC827 and H1650 are FHIT-expressing lines, and H1299 and H460 are FHIT-deficient lines. GAPDH was used as the internal control. **b** Schematic illustration of highly selective inhibitor library screening to discover synthetic lethal candidates for FHIT deficiency. **c** The scatter plot presents the screening results as log10-IC_50_ values of the inhibitors. Inhibitors with an SI (parental-IC_50_/*FHIT*^−/−^-IC_50_) greater than two were considered synthetic lethal drugs for FHIT defects and are highlighted with colored dots. **d**–**f** Cell confluence was acquired from the Incucyte Zoom and normalized to that of the control group to validate the synthetic lethal effects of GSK3β inhibition. HCC827 (parental and *FHIT*^−/−^), H460 (*FHIT*^*OE*^ and parental) and H1299 (*FHIT*^*OE*^ and parental) cell pairs were treated with different doses of CHIR99021 for three days. Representative images of HCC827 (**g**), H460 (**h**) and H1299 (**i**) cells treated with the indicated concentrations of CHIR99021 for three days; scar bar = 50 μm. **j** Representative images of a colony formation assay in HCC827-FHIT isogenic cells. The cells were treated with 2.5 μM or 10 μM CHIR99021 (CHIR) for 10 days and stained with crystal violet. **k** Effects of CHIR99021 on the growth rate of HCC827 FHIT-isogenic cell pairs. The cells were incubated with 10 μM CHIR99021, and an IncuCyte Zoom was used to capture images of the cells at different time points to calculate cell confluence. The data are presented as the mean ± SD of each group. The statistical difference between the two groups was analyzed by two-way ANOVA. All FHIT-deficient cells are marked in red.

To investigate the synthetic lethal targets of FHIT in lung cancer, we established FHIT-isogenic lung cancer cell pairs via CRISPR/Cas9 gene knockout (KO) and lentiviral FHIT overexpression (OE). FHIT wild-type lung cancer cell lines, including HCC827 and H1650, were used to generate FHIT-KO clones, and FHIT-null cell lines, including H1299 and H460, were used to generate FHIT-OE clones (Supplementary Fig. 1a). The established KO cell lines were verified via RFP expression, single guide RNA (sgRNA) target site PCR, DNA sequencing and Western blotting of FHIT expression (Fig. 1a, Supplementary Fig. 1b–e), and the OE cell lines were verified via qPCR and Western blotting of FHIT expression (Fig. 1a, Supplementary Fig. 1f). These isogenic cell pairs were used for synthetic lethal target screening and validation.

We first used the HCC827 FHIT-isogenic cell pair (parental FHIT wild-type cells and *FHIT*^−/−^ cells) for synthetic lethal drug screening with a highly selective inhibitor library containing 318 specific small molecule inhibitors targeting various cellular druggable proteins. The drug screen was conducted in an 8-dose titration format in 384-well plates to obtain IC_50_ values for individual drugs against the parental and *FHIT*^−/−^ cells (Fig. 1b). Differential drug sensitivity between *FHIT*^−/−^ and the parental cells was calculated via the selectivity index (SI = IC_50_ for parental cells/IC_50_ for *FHIT*^−/−^ cells), and drugs with SI values greater than 2 were selected as hits (Fig. 1c). The identified hits included the glycogen synthase kinase 3 beta (GSK3β) inhibitor CHIR99021, the checkpoint kinase 1 (CHK1) inhibitor LY2603618, the sphingosine kinase (SPHK) inhibitor SKI II, and the Rac GTPase inhibitor NSC23766 (Fig. 1c).

Since the GSK3β inhibitor CHIR99021 was found to be the top synthetic lethal candidate drug, we studied the synthetic lethality between FHIT and GSK3β in greater detail. To validate the screening results, we examined the effect of CHIR99021 on cell growth in three FHIT-isogenic cell pairs, including FHIT-KO pairs (HCC827 parental vs. *FHIT*^−/−^ cells) and FHIT-OE pairs (H460 *FHIT*^*OE*^ vs. H460 parental cells and H1299 *FHIT*^*OE*^ vs. H1299 parental cells). FHIT-deficient lung cancer cells were more sensitive to the GSK3β inhibitor than FHIT-proficient cells were in all three FHIT-isogenic pairs tested (Fig. 1d–i). Moreover, the GSK3β inhibitor resulted in greater sensitivity in FHIT-deficient cells as determined by colony formation and real-time cell growth rate measurements (Fig. 1j, k).

**Fig. 2:**
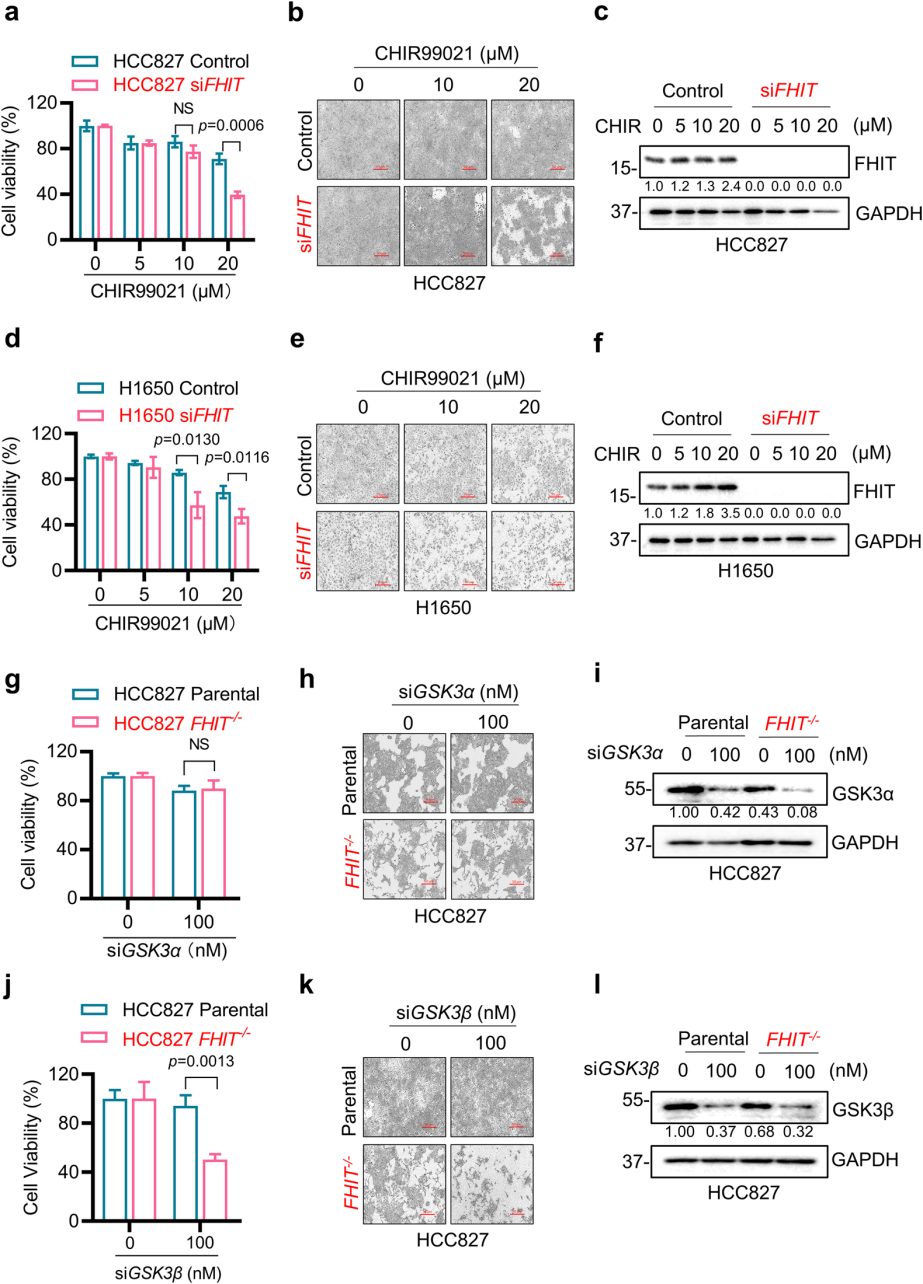
Validation of synthetic lethality by silencing FHIT or GSK3β in lung cancer cells. **a** Synthetic lethality in FHIT-knockdown HCC827 cells treated with CHIR99021 (CHIR). After HCC827 cells were transfected with or without 200 nM FHIT siRNA for 48 h, they were incubated with CHIR99021 (0, 5, 10, or 20 μM) for an additional three days. Cell viability was assessed via the Alamar blue assay. The data are presented as the mean ± SD of each group. Unpaired two-tailed Student’s *t* tests were used to determine the significance of the differences between two groups. **b** Representative cell images of **a**, scale bar = 50 μm. **c** Western blot analysis of FHIT protein knockdown in **a**. **d** Synthetic lethality in FHIT-knockdown H1650 cells treated with CHIR99021. After H1650 cells were transfected with or without 200 nM FHIT siRNA for 48 h, they were incubated with CHIR99021 (0, 5, 10, or 20 μM) for an additional three days. Cell viability was assessed via the Alamar blue assay. The data are presented as the mean ± SD of each group. Unpaired two-tailed Student’s t tests were used to determine differences between two groups. **e** Representative cell images of **d**, scale bar = 50 μm. **f** Western blot analysis of FHIT protein knockdown in **d**. **g**‒**l** Synthetic lethality of GSK3β knockdown in HCC827 *FHIT*^−/−^ cells. After HCC827 parental and *FHIT*^−/−^ cells were transfected with or without 100 nM GSK3α (**g**) or GSK3β (**j**) siRNA for 72 h, cell viability was determined via the Alamar blue assay. The data are presented as the mean ± SD of each group. Unpaired two-tailed Student’s *t* tests were used to determine the significance of the differences between two groups. NS denotes not significant. Representative cell images **h**, **k** of **g** and **j**, scale bar = 50 μm. Western blotting was used to detect the knockdown of the GSK3α (**i**) or GSK3β (**l**) proteins in **g** and **j**. GAPDH was used as the internal control. All FHIT-deficient cells are marked in red.

To verify that the selective antiproliferative effect of CHIR99021 in FHIT-deficient lung cancer cells was due to the synthetic lethal interaction between FHIT and GSK3β rather than the compound’s off-target effect, we silenced FHIT or GSK3β with specific siRNAs in lung cancer cells and measured cell viability. In HCC827 or H1650 FHIT wild-type cells, FHIT silencing led to cellular hypersensitivity to the GSK3β inhibitor (Fig. 2a–f). Furthermore, the silencing of GSK3α did not result in differential sensitivity in the FHIT-isogenic cell pair, whereas GSK3β silencing significantly inhibited the viability of *FHIT*^−/−^ cells (Fig. 2g–l). These data demonstrated that GSK3β is a potential synthetic lethal target of FHIT in lung cancer cells.

### Inhibition of GSK3β promotes DNA damage and apoptosis in FHIT-deficient lung cancer cells

**Fig. 3:**
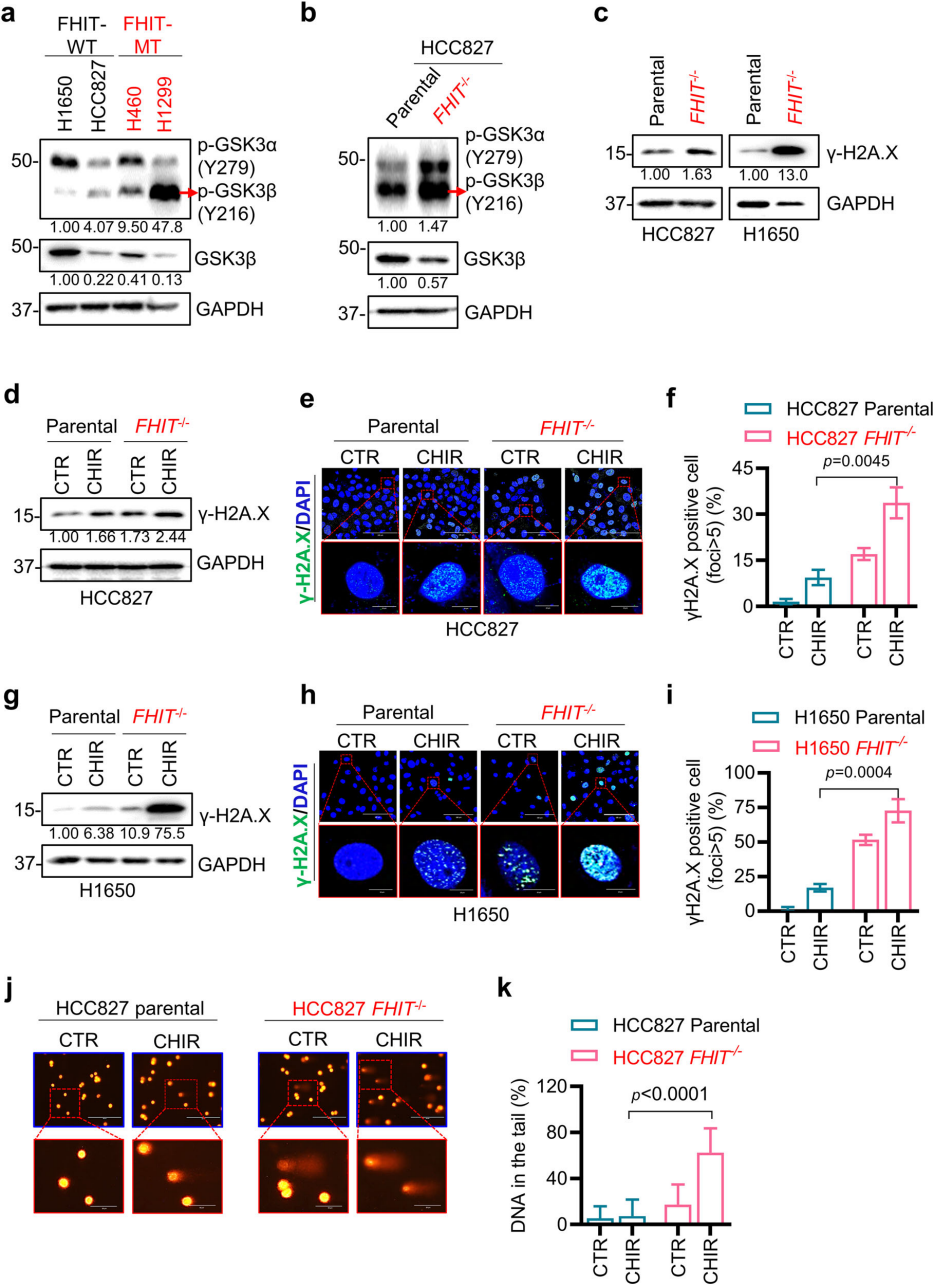
GSK3β inhibition induces DNA–double-strand breaks in lung cancer cells. **a** Western blotting was used to compare the protein levels of GSK3β and p-GSK3α/β (Y279/Y216) in four lung cancer cell lines, FHIT-expressing (H1650 and HCC827) and FHIT-defective (H460 and H1299) lines. **b** Western blot analysis of the protein levels of GSK3β and p-GSK3α/β (Y279/Y216) in the HCC827 FHIT-isogenic cell pair. **c** Western blot analysis of γ-H2A. X protein levels in the HCC827 and H1650 FHIT-isogenic cell pairs. **d**–**i** Effects of GSK3βi on DNA double-strand breaks in HCC827 and H1650 FHIT isogenic cells. The cells were treated with or without 20 μM CHIR99021 for 24 h, and the protein level of γ-H2A. X was examined by Western blotting (**d**, **g**), and the in situ foci of γ-H2A. X were detected via immunofluorescence staining, as shown in the fluorescence images; scale bar = 100 μm. Inset images (red squares) are enlarged and shown below each panel; scale bar = 10 μm. The nuclei were stained with DAPI (**e**, **h**), and the number of cells containing more than five γ-H2A. X foci in each group were recorded and quantified via ImageJ with a threshold (setting a fixed value: 74, 129) to distinguish positive γ-H2A. X foci from the background staining (**f**, **i**). The data are presented as the mean ± SD of each group. The difference between two groups was determined by an unpaired two-tailed Student’s *t* test. **j**, **k** Comet assay to test DNA damage in cells upon GSK3βi treatment. HCC827 FHIT-isogenic cells were treated with or without 20 μM CHIR99021 for 24 h to conduct an alkaline comet assay, and representative images from the comet assay are shown; scale bar = 200 μm. Inset images (red squares) are enlarged and shown below each panel; scale bar = 50 μm. Representative images (**j**) were acquired via an Evos microscope (Life Technology), and the percentage of DNA in the tail (**k**) was analyzed via the ImageJ component Open Comet. The data are presented as the mean ± SD of each group. The significance of the difference between two groups was determined by an unpaired two-tailed Student’s t test. GAPDH was used as the internal control. All FHIT-deficient cells are marked in red.

To explore the underlying mechanism of the observed synthetic lethality induced by GSK3β inhibition, we first compared the basal expression levels of GSK3β in FHIT-expressing and FHIT-mutant lung cancer cell lines. An increased level of p-GSK3β (Tyr216), an active version of GSK3β, was observed in both natural FHIT-mutant cells (Fig. 3a) and FHIT-KO cells (Fig. 3b), indicating that FHIT loss activated GSK3β. These results indicate that FHIT and GSK3β may participate in certain common biological processes. The main cause of FHIT loss is smoke or genotoxic stress-induced chromosomal damage at fragile sites. Conversely, FHIT is known to play an important role in maintaining genomic stability and integrity^19^. GSK3β also plays an important role in the DNA damage response signal by participating in DNA base excision repair and double-strand break repair^30,31^.

These findings led us to hypothesize that DNA damage and repair pathways could be common downstream pathways of both FHIT and GSK3β. Indeed, the level of the DNA damage marker γ-H2A. X was elevated in FHIT-deficient lung cancer cells (Fig. 3c). The accumulation of DNA damage in cells leads to a cellular response for DNA damage repair, a protective mechanism in response to damaged DNA where GSK3β acts as one of the primary proteins involved, which could explain the upregulation of p-GSK3β (Try216) in FHIT-deficient cells. Treatment of HCC827 FHIT-deficient cells with a GSK3β inhibitor further increased the level of γ-H2A. X (Fig. 3d). We also observed that FHIT-deficient HCC827 cells treated with a GSK3β inhibitor presented significantly increased γ-H2A. X nuclear foci formation (Fig. 3e, f). Similar results were observed in H1650 FHIT isogenic cells treated with a GSK3β inhibitor (Fig. 3g–i). Finally, we conducted a comet assay (single-cell gel electrophoresis) to detect DNA strand breaks in cells treated with a GSK3β inhibitor. Compared with parental cells, FHIT-deficient cells presented slightly more DNA strand breaks (Fig. 3j, k). GSK3β inhibitor treatment significantly increased the number of DNA strand breaks in HCC827 *FHIT*^−/−^ cells, resulting in longer DNA tails (Fig. 3j) and a greater percentage of DNA in the tails (Fig. 3k). These data suggest that FHIT loss elevates DNA damage in cells and activates the cellular DNA damage repair response, including that of GSK3β. Inhibition of GSK3β blocks a part of the DNA damage repair pathway, promoting high levels of DNA strand breaks in FHIT-deficient cells.

**Fig. 4:**
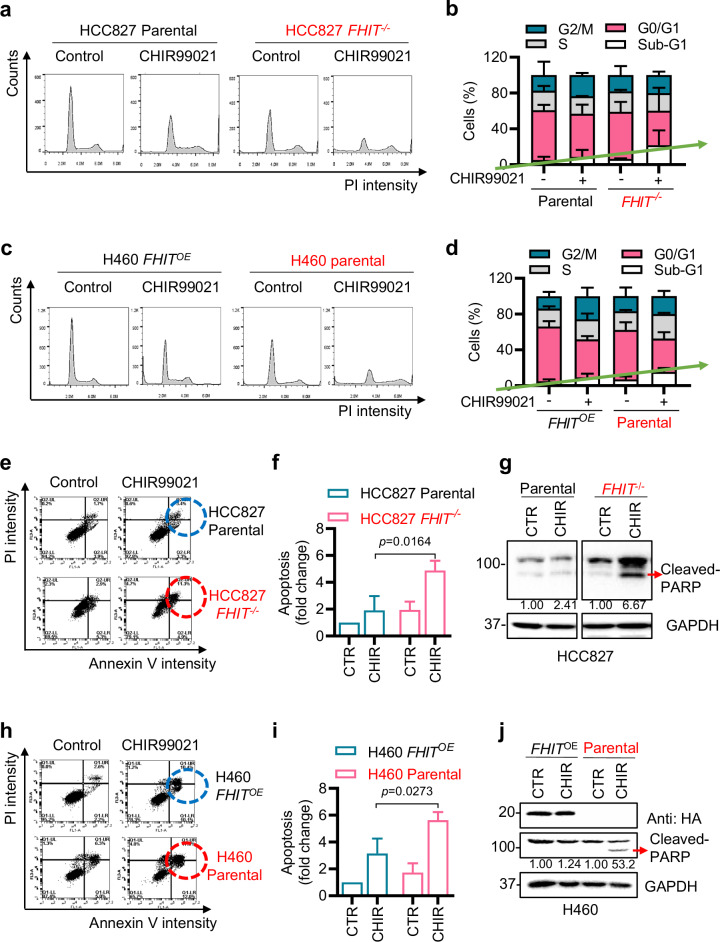
GSK3β inhibition increases the sub-G1 population and apoptosis in FHIT-deficient lung cancer cells. **a**–**d** Effects of GSK3βi on the cell cycle in HCC827 and H460 lung cancer cell pairs. The cells were treated with 20 μM CHIR99021 for 48 h, stained with propidium iodide (PI) to characterize the DNA content and detected via flow cytometry, as shown in the histogram chart (**a**, **c**). The percentages of cells distributed in the sub-G1, G0/G1, S, and G2/M phases are displayed in the bar chart (**b**, **d**). **e**–**j** Effect of GSK3βi on apoptosis in HCC827 and H460 lung cancer cell pairs. The cells were treated with CHIR99021 at 20 μM for 48 h, stained with FITC-Annexin V/PI and analyzed by flow cytometry, as shown in the scatterplot, in which the Y-axis represents the PI intensity and the X-axis represents the Annexin V intensity (**e**, **h**). The quantitative data of apoptotic cells from **e** and **h** are shown in the bar charts **f** and **i**. The data are presented as the mean ± SD of each group. The significance of the difference between two groups was determined by an unpaired two-tailed Student’s t test. **g**, **j** Western blot analysis of the protein levels of the apoptotic marker cleaved-PARP in the HCC827 and H460 cell pairs after treatment with 20 μM CHIR99021 for 48 h. GAPDH was used as the internal control. All FHIT-deficient cells are marked in red.

The accumulation of DNA damage often leads to cell cycle arrest, a status of autonomous or passive slowing down of cell division to earn enough time for DNA repair^32^. However, if the amount of damaged DNA overwhelms the capacity of the repair system, apoptotic cell death is initiated^33^. To analyze the correlation between the DNA damage response and synthetic lethal cell death induced by the GSK3β inhibitor, the cell cycle and apoptosis were measured in FHIT-isogenic cells treated with the GSK3β inhibitor. CHIR99021 markedly elevated the sub-G1 population in FHIT-deficient HCC827 (Fig. 4a, b) and H460 cells (Fig. 4c, d). Annexin-V staining and PARP Western blots also revealed that the GSK3β inhibitor CHIR99021 significantly increased apoptosis in FHIT-deficient lung cancer cells (Fig. 4e–j). These results suggested that inhibition of GSK3β selectively induced apoptosis in FHIT-deficient cells following increased DNA strand breaks.

### The GSK3β-dependent DNA double-strand break repair pathway is activated in FHIT-deficient lung cancer cells

**Fig. 5:**
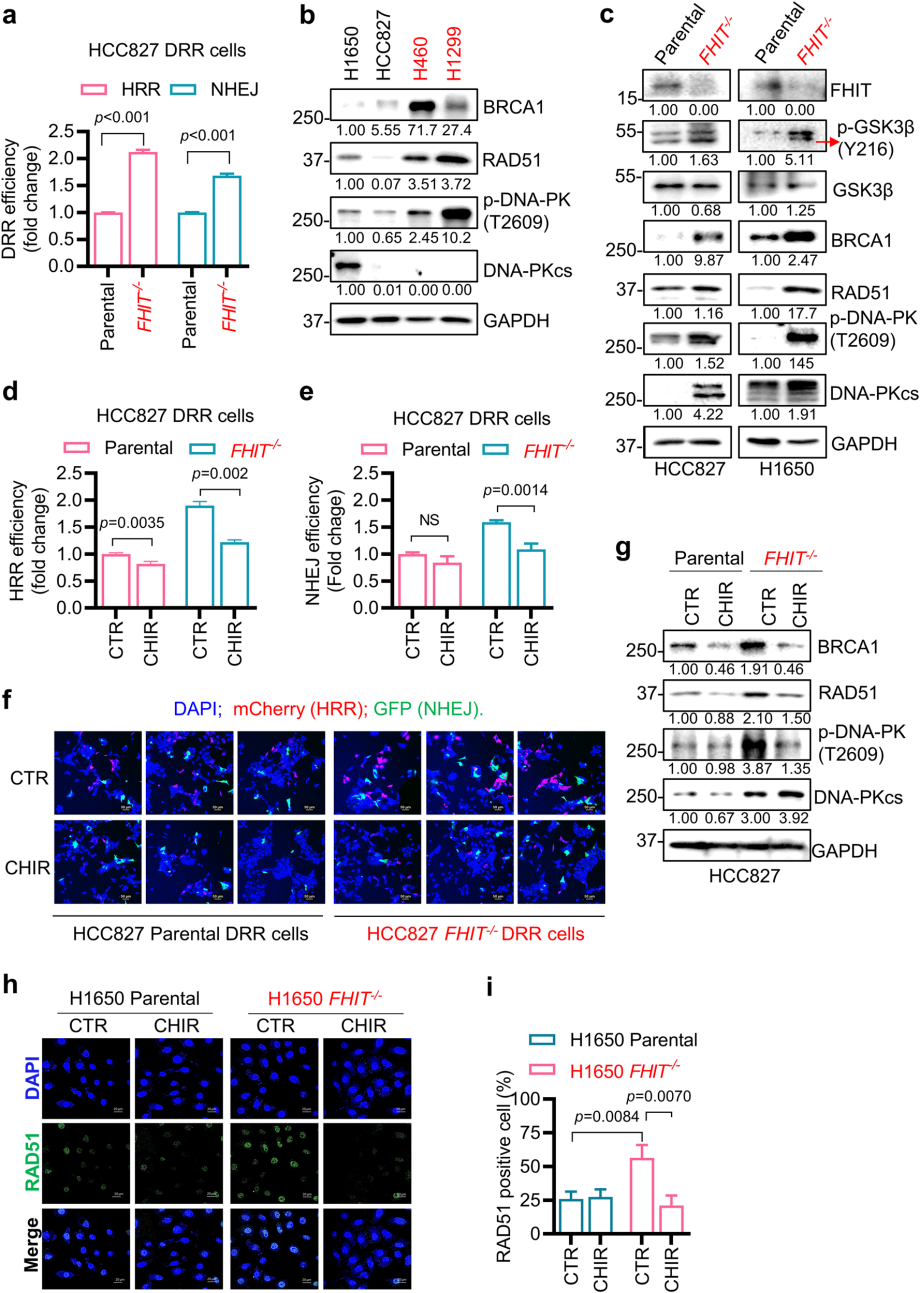
Inhibition of HRR and NHEJ in lung cancer cells by a GSK3β inhibitor. **a** Basal efficiency of HRR and NHEJ in HCC827 FHIT-isogenic DRR cells. Twenty-four hours after the transfection of HCC827 FHIT-isogenic DRR cells with pCBASceI and pCAGGS DRR mCherry Donor EF1a BFP plasmids, the transfected cells were analyzed via flow cytometry to quantify the efficiency of HRR (represented by the ratio of mCherry) and NHEJ (represented by the ratio of GFP). The data are presented as the mean ± SD of each group. The difference between two groups was determined by an unpaired two-tailed Student’s *t* test. **b** Basal expression of the critical factors involved in HRR and NHEJ in FHIT-expressing and FHIT-defective lung cancer cells. H1650, HCC827, H460 and H1299 cells were seeded into a 12-well plate for 24 h, and the protein levels of BRCA1, RAD51, P-DNA-PK (T2609) and DNA-PKcs were measured via Western blotting. **c** Basal expression of the critical factors involved in HRR and NHEJ in FHIT-isogenic lung cancer cell pairs. HCC827 and H1650 FHIT-isogenic cells were seeded into a 12-well plate for 24 h, the protein levels of BRCA1, RAD51, P-DNA-PK (T2609) and DNA-PKcs were measured via Western blotting. **d**–**f** Effects of GSK3βi on the efficiency of HRR and NHEJ. After HCC827 FHIT-isogenic DRR cells were transfected with pCBASceI and pCAGGS DRR mCherry Donor EF1a BFP plasmids for 24 h, the transfected cells were incubated with or without 20 μM CHIR99021 for 24 h. The efficiency of HRR (represented by the ratio of mCherry) and NHEJ (represented by the ratio of EGFP) was analyzed via flow cytometry (**d**, **e**). The data are presented as the mean ± SD of each group. The difference between two groups was determined by an unpaired two-tailed Student’s t test. NS denotes not significant. Representative cell fluorescence images (**f**) were also captured; scale bar = 50 μm. The nuclei were stained with Hoechst 33342. **g** Effects of GSK3βi on the expression of critical factors involved in HRR and NHEJ signaling. The protein levels of BRCA1, RAD51, P-DNA-PK (T2609) and DNA-PKcs were measured by Western blot in HCC827 FHIT-isogenic cells after treatment with or without 20 μM CHIR99021 for 24 h. **h**, **i** Immunofluorescence staining was used to detect the in situ foci of RAD51 in H1650 FHIT-isogenic cells after treatment with or without 20 μM CHIR99021 for 24 h, as shown in the representative images (**h**), scale bar = 20 μm. The number of RAD51 foci-positive cells in each group was recorded and quantified (**i**). The data are presented as the mean ± SD of each group. The difference between two groups was determined by an unpaired two-tailed Student’s *t* test. GAPDH was used as the internal control. All FHIT-deficient cells are marked in red.

To investigate the mechanism underlying the activation of the DNA damage response in FHIT-deficient lung cancer cells, DNA damage repair (DRR) cell lines were constructed^25^. *FHIT*-isogenic HCC827 cells and FHIT-deficient H460 lung cancer cells were stably transfected with a reporter plasmid containing the I-SceI recognition sequence with EGFP, and successful reporter expression was verified via PCR amplification of the neomycin resistance gene in DRR cells (Supplementary Fig. 2a–c). The I-SceI enzyme plasmid and the homologous recombination repair (HRR) donor plasmid containing mCherry were transiently transfected into DRR cells to detect the nonhomologous end joining (NHEJ) or HRR repair efficiency. I-SceI-mediated DNA double-strand breaks (DSBs) can be repaired either by the cellular NHEJ or HRR system, and each DSB repair activity can be visualized by EGFP or mCherry expression in reporter cells. *FHIT*^−/−^ HCC827 cells presented significantly greater HRR activity than FHIT-wild-type HCC827 cells did (Fig. 5a). Similarly, FHIT-deficient H460 cells presented greater HRR activity than FHIT-wild-type HCC827 cells did (Supplementary Fig. 3a). NHEJ activity was also greater, albeit marginally so, in *FHIT*^−/−^ HCC827 and FHIT-deficient H460 cells than in FHIT-wild-type HCC827 cells (Fig. 5a and Supplementary Fig. 3a).

We further analyzed representative HRR and NHEJ marker proteins, including the levels of BRCA1 and RAD51 (for HRR) and the level of phosphorylated DNA-PKcs at T2609 (for NHEJ), in four lung cancer cell panels with different FHIT statuses and two FHIT-isogenic cell pairs. All FHIT-deficient lung cancer cells presented increased expression levels of BRCA1 and RAD51 and increased levels of phosphorylated DNA-PKcs (Fig. 5b, c). These data indicate that FHIT-deficient lung cancer cells have increased HRR and NHEJ activities compared with their FHIT-wild-type counterparts. Further analysis of lung cancer patient cohorts from The Cancer Genome Atlas (TCGA) datasets revealed that FHIT protein levels were negatively associated with BRCA1 and RAD51 levels in patients (analyzed by Linked Omics, https://linkedomics.org/admin.php) (Supplementary Fig. 4a, b). These data further strengthened our notion that FHIT deficiency activates the HRR DSB repair pathway in lung cancer cells.

We next examined whether GSK3β was involved in the activation of the DSB repair pathway in lung cancer cells. The activity of DSB repair pathways, especially HRR activity, was increased in *FHIT*^−/−^ HCC827 and FHIT-deficient H460 cells and was significantly suppressed by treatment with the GSK3β inhibitor CHIR99021 (Fig. 5d, e and Supplementary Fig. 3b, c). Representative mCherry (HRR) and EGFP (NHEJ) images of the reporter cell lines treated with or without the GSK3β inhibitor are shown in Fig. 5f and Supplementary Fig. 3d, indicating that GSK3β inhibition blocked DSB repair activities in lung cancer cells.

We further investigated the effects of the GSK3β inhibitor on the HRR and NHEJ signaling pathways in FHIT-wild-type and FHIT-deficient lung cancer cells. The elevated BRCA1, RAD51 and phosphorylated DNA-PKcs levels in *FHIT*^−/−^ HCC827 and FHIT-deficient H460 cells were obviously downregulated by CHIR99021 (Fig. 5g and Supplementary Fig. 3e). Furthermore, the GSK3β inhibitor significantly decreased the number of nuclear RAD51 foci (Fig. 5h) and the percentage of RAD51-positive cells (Fig. 5i; Supplementary Fig. 3f) in FHIT-deficient cells. These results suggest that the GSK3β-dependent DSB repair pathway is activated in FHIT-deficient lung cancer cells and provide a scientific basis for the mechanism underlying GSK3β inhibitor-induced DNA damage and synthetic lethality in FHIT-deficient lung cancer cells.

### FHIT regulates HRR protein stability, and GSK3β regulates HRR gene transcription

**Fig. 6:**
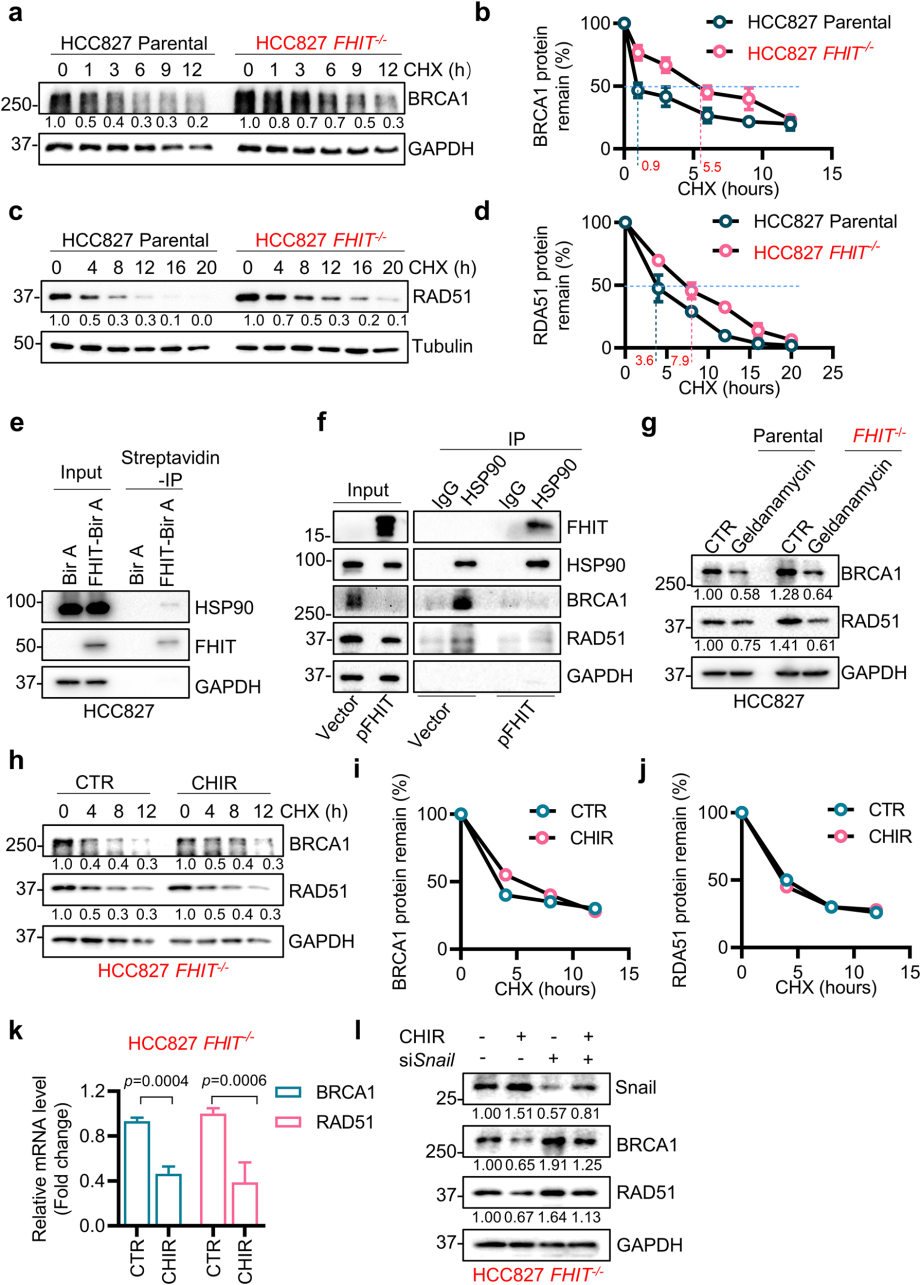
FHIT deficiency and GSK3β inhibition regulate BRCA1 and RAD51 at different steps. **a**–**d** Effect of FHIT deficiency on the protein stability of BRCA1 and RAD51. After HCC827 parental and HCC827 *FHIT*^−/−^ cells were incubated with 50 μg/mL CHX for the indicated times, Western blotting was performed to determine the protein levels of BRCA1 (**a**) and RAD51 (**c**), and ImageJ software was used to quantify the percentage of BRCA1 (**b**) and RAD51 (**d**) protein remaining. **e**, **f** HSP90 interacts with FHIT and BRCA1/RAD51. BioID was used to detect the interaction between FHIT and HSP90 in HCC827 cells (**e**), and HSP90 IP was used to analyze the interactions between HSP90 and FHIT, BRCA1, and RAD51 (**f**). **g** Western blot analysis of the protein levels of BRCA1 and RAD51 in HCC827 FHIT-isogenic cells treated with 125 nM geldanamycin for 48 h. GAPDH was used as the internal control. **h**–**j** Effect of a GSK3β inhibitor on the protein stability of BRCA1 and RAD51. After the cells were treated with or without 20 μM CHIR99021 for 30 min and then incubated with 50 μg/mL CHX for the indicated times, Western blotting was performed to determine the protein levels of BRCA1 and RAD51 in HCC827 *FHIT*^−/−^ cells (**h**), and ImageJ software was used to quantify the percentage of remaining BRCA1 and RAD51 proteins (**i**, **j**). **k** Effect of a GSK3β inhibitor on the mRNA levels of BRCA1 and RAD51. After the HCC827 *FHIT*^−/−^ cells were treated with or without 20 μM CHIR99021 for 12 h, RT‒qPCR was used to examine the mRNA levels of BRCA1 and RAD51. The data are presented as the means ± SDs of each group. The difference between two groups was determined by an unpaired two-tailed Student’s *t* test. **l** The effect of si*Snail* on CHIR99021-induced BRCA1 and RAD51 downregulation. HCC827 *FHIT*^−/−^ cells were transfected with 200 nM Snail siRNA for 72 hours and then treated with 20 μM CHIR99021 for 24 h. The cell lysates were analyzed via Western blotting for Snail, BRCA1 and RAD51. GAPDH was used as the internal control. All FHIT-deficient cells are marked in red.

We observed that FHIT and GSK3β regulate the protein expression of BRCA1 and RAD51 in HRR, and the phosphorylation status of DNA-PKcs in NHEJ. To further investigate the mechanisms by which FHIT and GSK3β regulate the levels of BRCA1 and RAD51, we first analyzed the changes in the stability of the BRCA1 and RAD51 proteins in FHIT-isogenic cells treated with the protein synthesis inhibitor cycloheximide (CHX). The half-lives of both the BRCA1 and RAD51 proteins were significantly longer in *FHIT*^−/−^ HCC827 cells than in parental cells (0.9 h vs. 5.5 h for BRCA1 and 3.6 h vs. 7.9 h for RAD51) (Fig. 6a–d). These data suggest that FHIT deficiency promotes the protein stability of BRCA1 and RAD51 in lung cancer cells. Since BRCA1 and RAD51 protein stability are known to be regulated by HSP90 and FHIT is known to bind to HSP90^34–36^, we speculated that HSP90 might be involved in the regulation of HRR protein stability by FHIT. We thus explored the interaction between HSP90 and the FHIT and HRR proteins and analyzed the role of the interaction in HRR protein stability. We first verified the interaction between FHIT and HSP90 in HCC827 and H1299 lung cancer cells (Fig. 6e and Supplementary Fig. 5a). We next tested the interaction between HSP90 and HRR proteins in FHIT-deficient cells. In *FHIT*^−/−^ HCC827 cells, HSP90 strongly binds to BRCA1 and RAD51 (Fig. 6f). However, when FHIT was re-expressed in the cells, the interaction between FHIT and BRCA1 or RAD51 was significantly weakened, and the total protein levels of the HRR proteins were thereby reduced (Fig. 6f). Geldanamycin, a small molecule HSP90 inhibitor, significantly reduced the protein levels of BRCA1 and RAD51 (Fig. 6g), and this reduction was partly reversed by cotreatment with the proteasome inhibitor MG132 (Supplementary Fig. 5b). These data suggest that FHIT regulates HRR protein stability through HSP90.

As we also observed that GSK3β inhibition reduced the levels of HRR proteins, we next explored the underlying mechanism of this regulation. Interestingly, the GSK3β inhibitor did not affect the half-lives of the BRCA1 and RAD51 proteins (Fig. 6h, j and Supplementary Fig. 6a–c) but significantly reduced their mRNA levels (Fig. 6k and Supplementary Fig. 6d), implying that GSK3β regulates the level of HRR proteins at the transcriptional level.

Since HRR gene expression has been reported to be regulated by several transcription factors, such as Snail, Slug and RELB/NF-κB^37,38^, we explored the potential involvement of these transcription factors in the regulation by GSK3β. We found that the GSK3β inhibitor increased Snail and Slug expression, and the silencing of Snail or Slug reversed the GSK3β inhibitor-induced decrease in BRCA1 and RAD51 levels in *FHIT*^−/−^ HCC827 cells (Fig. 6l and Supplementary Fig. 6e). This result was consistent with previous observations that Snail or Slug act as transcriptional repressors of BRCA1 and that the GSK3β pathway regulates upstream Snail/Slug-mediated transcriptional regulation of HRR genes^37^. RELB silencing did not rescue the effect of the GSK3β inhibitor on BRCA1 and RAD51, excluding the possibility of the involvement of RELB/NF-κB in the regulation of HRR gene transcription by GSK3β (Supplementary Fig. 6f).

### HRR blockade plays a major role in GSK3β inhibitor-induced synthetic lethality in FHIT-deficient lung cancer cells

**Fig. 7:**
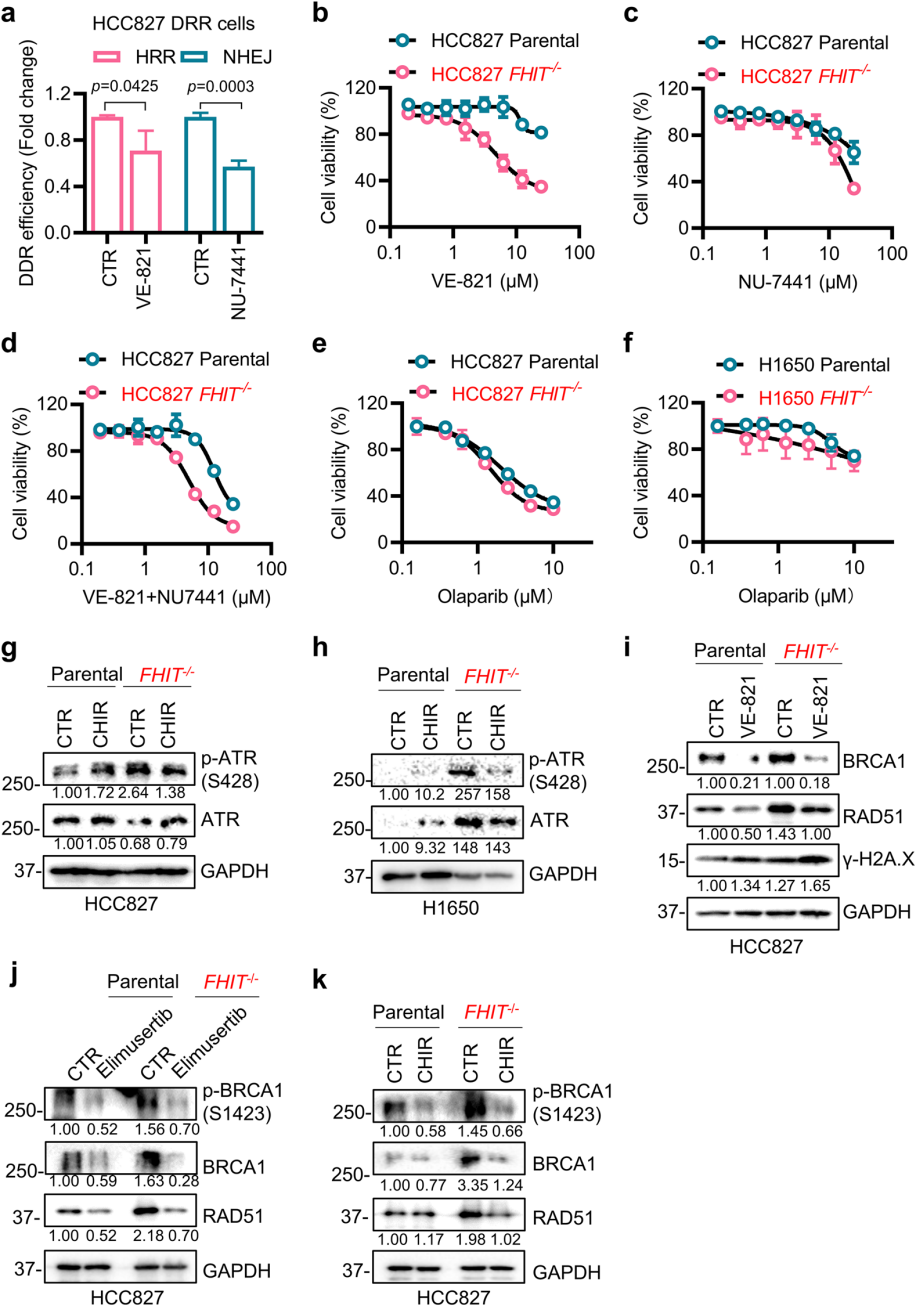
HRR blockade contributes to synthetic lethality in FHIT-deficient lung cancer cells. **a** Effect of VE-821 on HRR efficiency and NU-7441 on NHEJ efficiency. After HCC827 DRR cells were transfected with pCBASceI and pCAGGS DRR mCherry Donor EF1a BFP plasmids for 24 h, the transfected cells were incubated with or without 5 μM VE-821 or 5 μM NU-7441 for 48 h. The efficiency of HRR (represented by the ratio of mCherry) and NHEJ (represented by the ratio of EGFP) was analyzed via flow cytometry. The data are presented as the mean ± SD of each group. The difference between two groups was determined by an unpaired two-tailed Student’s *t* test. Synthetic lethality in HCC827 FHIT isogenic cells treated with VE-821 or NU-7441. After the cells were treated with VE-821 (**b**), NU-7441 (**c**) or both (**d**) at different concentrations for three days, cell viability was tested via the Alamar blue assay. **e**, **f** Effect of the PARP inhibitor olaparib on cell viability. HCC827 and H1650 FHIT isogenic cells were treated with various concentrations of olaparib for three days, and cell viability was tested via the Alamar blue assay. Western blotting was used to detect the protein levels of ATR and p-ATR (S428) in HCC827 (**g**) and H1650 (**h**) FHIT-isogenic cells treated with or without 20 μM CHIR99021 for 24 h. **i** Western blotting was used to detect the protein levels of BRCA1, RAD51 and γ-H2A. X in HCC827-isogenic cells treated with or without 10 μM VE-821 for 24 h. **j**, **k** Effects of limusertib and CHIR99021 on p-BRCA1 (s1423), BRCA1, and RAD51 protein levels. The cells were treated with 0.2 μM elimusertib and 20 μM CHIR99021 for 24 h, and Western blotting was used to detect protein expression. GAPDH was used as the internal control. All FHIT-deficient cells are marked in red.

Since both the HRR and NHEJ repair systems are activated in FHIT-deficient cells and since GSK3β inhibition can suppress these pathways, we sought to define which DSB repair pathway plays a major role in the synthetic lethality. We used the ATR inhibitor VE-821 and the DNA-PK inhibitor NU-7441 as inhibitors of HRR and NHEJ, respectively, and analyzed their synthetic lethal effects on FHIT-deficient lung cancer cells. We first confirmed that VE-821 and NU-7441 inhibited HRR and NHEJ, respectively, in HCC827 DRR cells (Fig. 7a). Our results revealed that the HRR inhibitor VE-821 had a strong synthetic lethal effect on HC827 *FHIT*^−/−^ cells, whereas NU-7441 did not (Fig. 7b, c). Moreover, the combination of the two drugs did not further increase the synthetic lethality of VE-821 alone, suggesting that HRR inhibition plays a major role in the synthetic lethality in FHIT-deficient lung cancer cells (Fig. 7d). Olaparib, an inhibitor of PARP involved in DNA single-strand break repair, also did not have a significant synthetic lethal effect on FHIT-deficient lung cancer cells (Fig. 7e, f).

We also tested the effects of geldanamycin, an inhibitor of HSP90 that has been shown to mediate FHIT-regulated HRR protein stability, on synthetic lethality in FHIT-deficient lung cancer cells. We observed that geldanamycin partially induced synthetic lethality with FHIT loss in lung cancer cells, accompanied by the downregulation of BRCA1 and RAD51 levels (Supplementary Fig. 7a, c). These results suggested that the synthetic lethal effect of the GSK3β inhibitor on FHIT-deficient cells was primarily mediated by the inhibition of HRR.

GSK3β is known to be involved in the DNA damage response by regulating the stability and activity of ATR^39^. We next analyzed the ATR status downstream of GSK3β in DNA damage response signaling in FHIT-isogenic cells. ATR phosphorylation was highly elevated in FHIT-deficient HCC827 and H1650 cells, and GSK3β inhibitor treatment significantly reduced the level of ATR phosphorylation in FHIT-deficient cells (Fig. 7g, h). Moreover, the ATR inhibitor VE-821 reduced the levels of BRCA1 and RAD51 and increased γ-H2A. X in FHIT-deficient lung cancer cells (Fig. 7i). Our data are partly in agreement with a previous report that BRCA1 and RAD51 are downstream effectors of ATR in the irradiation-induced DNA damage response pathway^40^.

Since the ATR inhibitor VE-821 also reduced total protein levels of BRCA1 and RAD51, similar to the GSK3β inhibitor, we wondered whether the ATR inhibitor-induced HRR protein downregulation was due to the inhibition of signaling pathways rather than the transcriptional regulation observed with the GSK3β inhibitor treatment. Given that ATR regulates BRCA1 phosphorylation^41,42^, we treated cells with another specific ATR inhibitor, elimusertib, and analyzed BRCA1 phosphorylation and HRR protein levels. The results revealed that the level of phospho-BRCA1 (S1423) was significantly reduced, followed by downregulation of total BRCA1 and RAD51 protein levels (Fig. 7j). A very similar result was observed upon treatment with the GSK3β inhibitor CHIR99021 (Fig. 7k). However, elimusertib had no effect on BRCA1 or RAD51 mRNA levels (Supplementary Fig. 8a), which is different from the effect observed with CHIR99021 treatment (Fig. 6k).

On the basis of these and earlier data, we hypothesized that the regulation of HRR protein levels by GSK3β is mediated by two distinct pathways: (1) GSK3β inhibition reduces BRCA1 phosphorylation and the total protein levels of BRCA1 and RAD51, and this effect is attributable to the inhibition of ATR phosphorylation and activity. (2) GSK3β inhibition reduces RAD51 and BRCA1 mRNA transcription by activating Snail/Slug transcription factors (transcription repressors for RAD51 and BRCA1). Our results also suggest that the regulation of HRR protein stability in FHIT-deficient cells involves two distinct pathways: (1) Regulation of the protein stability of RAD51 and BRCA1 by the competitive interaction between HSP90 and FHIT. (2) Long-term activation of the ATR pathway by FHIT loss-induced DNA DSBs, which in turn increases BRCA1 phosphorylation and HRR protein stability.

Since FHIT functions as a diadenosine triphosphate (Ap3A) hydrolase involved in nucleotide metabolism, one could question whether the diadenosine triphosphate hydrolase activity of FHIT is crucial for HRR protein stability. We introduced wild-type FHIT and FHIT-H96N, a hydrolase-dead mutant version^43^, into FHIT-deficient lung cancer cells and observed the effect of FHIT overexpression on HRR protein stability. The results revealed that both wild-type and hydrolase-dead mutant FHIT successfully reduced the BRCA1 and RAD51 protein levels in *FHIT*^−/−^ lung cancer cells (Supplementary Fig. 8b), suggesting that the Ap3A hydrolase activity of FHIT is not crucial for HRR protein stability or DNA damage repair signaling. This result is in line with earlier studies showing that the Ap3A hydrolase activity of FHIT is not crucial for its tumor suppressive functions^43^.

### GSK3β inhibitor selectively suppresses *FHIT*^−/−^ tumor growth in vivo

**Fig. 8:**
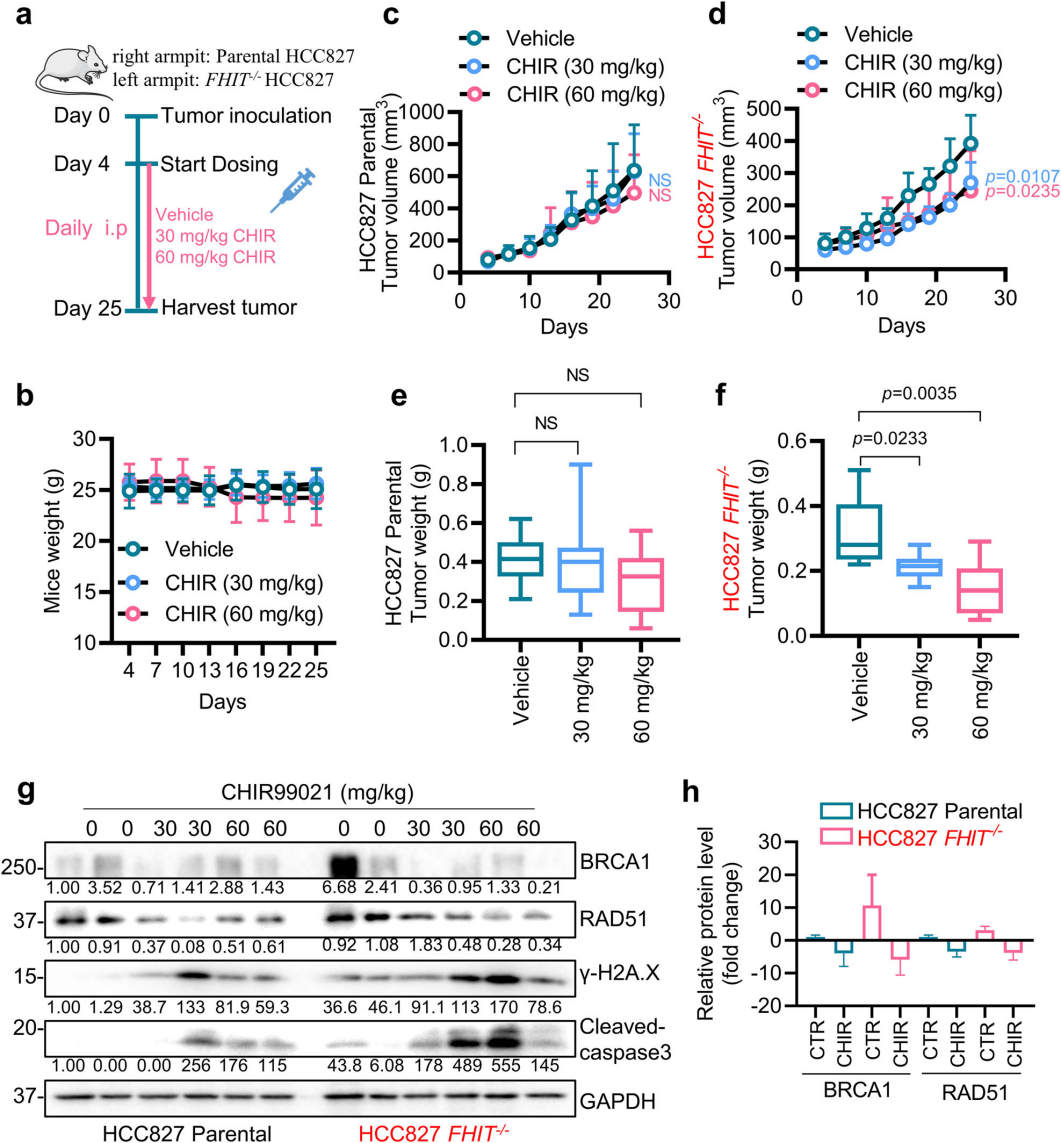
A GSK3β inhibitor selectively inhibits *FHIT*^−/−^ tumor growth in vivo. **a** Schematic diagram of the mouse tumor xenograft experiment. The mice injected with HCC827 parental and HCC827 *FHIT*^−/−^ cells were divided into 3 groups (8 mice/group). On the fourth day after tumor inoculation, the mice were treated with vehicle, 30 mg/kg CHIR99021 or 60 mg/kg CHIR99021 daily via intraperitoneal injection for 21 days. On the 25th day after tumor inoculation, all of the mice were euthanized, and the tumor tissues were collected. **b** The body weight of each mouse was measured every three days. The tumor volume of HCC827 parental (**c**) and HCC827 *FHIT*^−/−^ (**d**) tumors in each mouse was measured every three days. The data are presented as the mean ± SD of each group. Two-way ANOVA was used to analyze the significance of the differences between the two groups. NS denotes not significant. The weights of the HCC827 parental (**e**) and HCC827 *FHIT*^−/−^ (**f**) tumors were measured after the tumors were harvested from each mouse. The data are presented as the mean ± SD of each group. The difference between two groups was determined by an unpaired two-tailed Student’s t test. NS denotes not significant. **g**, **h** Western blot analysis of the protein levels of BRCA1, RAD51, and γ-H2A. X, and cleaved caspase-3 in tumor tissues randomly selected from the vehicle, 30 mg/kg CHIR99021 and 60 mg/kg CHIR99021 groups. All FHIT-deficient cells are marked in red.

To evaluate the in vivo therapeutic effect of the GSK3β inhibitor on lung cancer with FHIT loss, we performed tumor xenograft experiments with parental and *FHIT*^−/−^ HCC827 lung cancer cells in athymic nude mice (Fig. 8a). We designed a two-dose regimen of CHIR99021 (30 mg/kg and 60 mg/kg) for mouse experiments on the basis of previous reports^44^. The mice were administered vehicle or CHIR99021 via intraperitoneal (i.p.) injection daily for 21 consecutive days. The tumor volume and body weight were measured every 3 days. At this dosage, all mice were active and showed no signs of toxicity from CHIR99021 (Fig. 8b). Both 30 mg/kg and 60 mg/kg CHIR99021 significantly reduced the tumor volume and wet weight of *FHIT*^−/−^ HCC827 tumor xenografts, whereas the drug did not have apparent antitumor effects on the parental HCC827 tumor xenografts (Fig. 8c–f). Analysis of tumor samples revealed that BRCA1 and RAD51 levels were elevated in FHIT-deficient lung cancer, and CHIR99021 treatment reduced their protein levels (Fig. 8g, h). Moreover, γ-H2A. X and cleaved-caspase 3 levels were largely increased in FHIT-deficient lung cancer cells treated with CHIR99021 (Fig. 8g). These results demonstrated that the GSK3β inhibitor induced synthetic lethality in FHIT-deficient lung cancer in vivo by inhibiting HRR and inducing genotoxic cell death.

## Discussion

Synthetic lethality is a unique strategy to target ‘undruggable’ tumor proteins, such as mutant or inactivated tumor suppressors. Since the successful clinical introduction of PARP inhibitors for the treatment of multiple BRCAness tumors, synthetic lethality has been recognized as a promising approach to treat tumors with various tumor suppressor mutations. FHIT is a fragile site tumor suppressor that is located within FRA3B, a chromosome fragile locus where chromosome deletions occur under conditions of replication stress or genotoxic stress^45^. Loss of FHIT is frequently observed in many types of cancers, especially lung cancers in smokers^6^. Since its discovery, numerous studies have been conducted to elucidate the molecular mechanisms of FHIT in cancer. However, the precise role of FHIT loss in lung cancer carcinogenesis and tumor development is not fully understood. As such, little progress has been made in exploiting the loss of FHIT for lung cancer targeted therapy development. In this study, we employed a synthetic lethal drug screening approach to target lung cancer with FHIT loss and identified GSK3β as a synthetic lethal partner of FHIT in lung cancer cells. The synthetic lethality of GSK3β and FHIT has been validated in multiple FHIT-isogenic cell panels and in tumor xenograft models in vivo. Our in-depth mechanistic study of the synthetic lethality of GSK3β-FHIT in lung cancer established the following model: (1) FHIT loss activates the DNA damage response, particularly the HRR repair pathway, following increased DNA damage. (2) The activation of the HRR pathway in FHIT-deficient cells is mediated by both the GSK3β-ATR-BRCA1-RAD51 signaling axis and HSP90-mediated BRCA1/RAD51 protein stability. (3) The activated HRR mitigated genotoxic damage and increased the cell survival threshold in FHIT-deficient cells. (4) Inhibition of the GSK3β or HRR pathway compromises DNA damage repair activity and facilitates genotoxic damage-induced cell death in FHIT-deficient cells. This study revealed that the GSK3β and HRR pathways are new targets for developing FHIT loss-associated targeted therapies for lung cancer in smokers.

GSK3β is an evolutionarily conserved serine/threonine protein kinase that regulates a variety of biological processes, including cell proliferation^46^, differentiation^47^, the cell cycle^48^ and neural development and plasticity^49^. In cancer, GSK3β is a crucial component of several oncogenic signaling pathways, such as the WNT/β-catenin, Hedgehog (HH), Notch and MYC pathways^50^. GSK3β has long been recognized as a tumor suppressor, as it suppresses WNT/β-catenin signaling and EMT^51,52^. However, increasing evidence suggests that dysregulation or aberrant expression of GSK3β facilitates tumor progression^53,54^. For example, GSK3β promotes the transcriptional activity of NF-κB through the IκB kinase complex, thereby maintaining the NF-κB-mediated tumor survival pathway^55^. GSK3β also cooperates with mTOR to regulate the activity of the p70 ribosomal protein S6 kinase 1 (S6K1) to support cell growth^56^. In addition, many recent studies have shown that GSK3β directly responds to DNA damage and participates in the activation of DNA repair pathways^31,37,57–60^. Inhibiting GSK3β could sensitize various cancers to chemotherapy and radiotherapy by disturbing DNA damage repair^31,61^. These observations are in line with our findings that GSK3β plays a key role in the activation of the DNA DSB repair pathway in FHIT-deficient lung cancer cells and that the GSK3β inhibitor sensitizes FHIT-deficient cells to genotoxic stress. DNA DSBs in eukaryotic cells are repaired primarily by two common mechanisms, HRR and NHEJ, to ensure genome stability^62^. We noted that FHIT loss activated both the HRR and NHEJ repair pathways in lung cancer cells and that the GSK3β inhibitor inhibited both pathways. However, synthetic lethality was caused mainly by the inhibition of HRR but not NHEJ. This was presumably because HRR was strongly activated in FHIT-deficient cells (>2- or 4-fold greater activation in FHIT-mutant cells than in FHIT-wild-type cells), whereas NHEJ was marginally activated in FHIT-deficient cells (<2- or 1.5-fold greater activation in FHIT-mutant cells than in FHIT-wild-type cells). These data suggest that FHIT loss primarily activates the HRR repair pathway to mitigate genotoxic damage and that the HRR pathway, in addition to GSK3β, could be a druggable target for lung cancers with FHIT loss.

The role of FHIT in the suppression of DNA damage and the maintenance of genome stability has been suggested in several studies. However, the precise mechanism by which FHIT loss increases DNA damage remains elusive. Pichiorri and colleagues reported that FHIT interacts with heat shock proteins (HSPs) and regulates stress responses^21^. FHIT also interacts with mitochondrial ferredoxin reductase and modulates the level of reactive oxygen species (ROS). They suggested that the lack of FHIT would affect the functions of these proteins that modulate genotoxic and oxidative stresses, increasing the amount of damaged DNA in cells. Saldivar et al. reported that FHIT regulates nucleotide balance by modulating thymidine kinase 1 expression and subsequently the thymidine triphosphate pool, thus inducing DNA replication stress and DNA breakage in FHIT-deficient cells^19^. The regulatory effect of FHIT on thymidine kinase was later attributed to its intrinsic enzyme activities, including Ap3A hydrolase activity and scavenger-decapping enzyme activity^63^. Although it is unclear which mechanism FHIT uses to prevent DNA damage, our study suggests that Ap3A hydrolase activity is not crucial for its function in the DNA damage response, supporting the former observations that the interaction of FHIT with partner proteins may play an important role in the DNA damage response. Our additional study revealed that FHIT-deficient cells have elevated ROS and γ-H2A. X levels, and the treatment of the cells with the antioxidant N-acetylcysteine significantly reduced γ-H2A. X levels in FHIT-deficient lung cancer cells (Supplementary Fig. 9a, d). These data indicate that FHIT interacts with mitochondrial proteins and modulates ROS levels, thus explaining oxidative stress-induced DNA damage in FHIT-deficient cells. We are currently investigating a genome-wide, FHIT-protein interaction analysis in lung cancer cells to uncover proteins and pathways that FHIT interacts with and modulates. This information will largely facilitate the elucidation of the functions of FHIT in tumor suppression in lung cancer and promote the identification of new druggable targets associated with FHIT loss.

In conclusion, this study revealed synthetic lethality between FHIT and GSK3β in lung cancer cells. We showed that a GSK3β inhibitor selectively inhibited FHIT-deficient lung cancer by inhibiting the ATR/BRCA1/RAD51 signaling axis and BRCA1/RAD51 transcription, leading to the inhibition of HRR and genotoxic cell death. Our study provides strong evidence that inhibiting the GSK3β and HRR pathways is a promising strategy for targeting FHIT loss in smokers with lung cancer.

## Supplementary information

Supplementary Information
