# Supplementary material for: Inhibition of GSK3β is synthetic lethal with FHIT loss in lung cancer by blocking homologous recombination repair
Source: Exp Mol Med. 2025 Jan 6;57(1):167–83. doi: 10.1038/s12276-024-01374-0 (PMC11799392; doi:10.1038/s12276-024-01374-0)
Supplement: Supplementary file 1 — Supplementary Information [file 12276_2024_1374_MOESM1_ESM.pdf]

Supplementary information

Inhibition of GSK3β is synthetic lethal with FHIT loss in lung cancer by blocking homologous recombination repair

Shishi Tao et al.

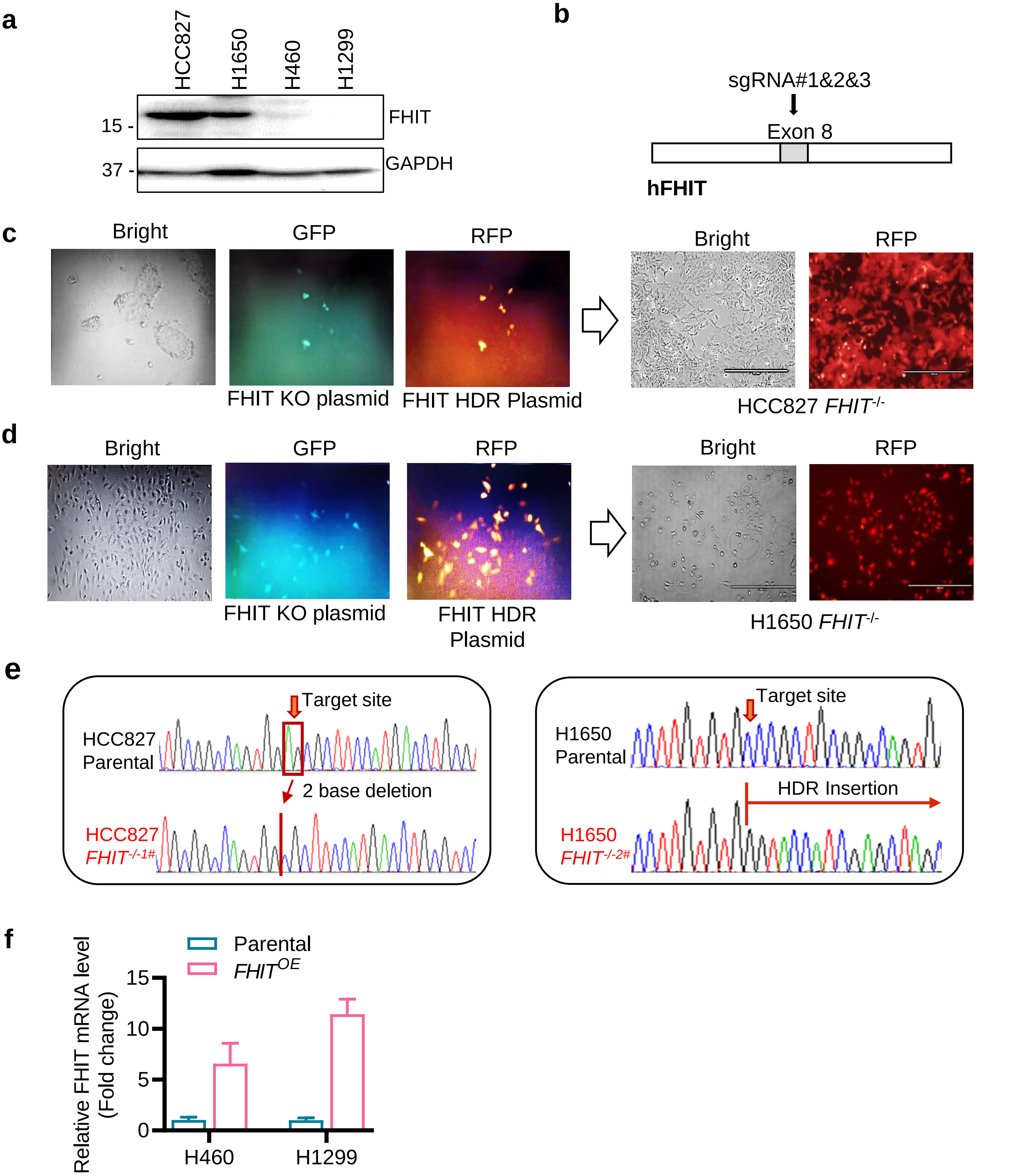

Supplementary Fig. 1. Generation of *FHIT*$^{-/-}$ and FHIT-overexpression (OE) cells. a Western blot to analyze the expression level of FHIT in different lung cancer cell lines. **b** Schematic representation of sgRNAs-targeted FHIT exons. **c, d** The GFP and RFP expression after the HCC827 (**c**) and H1650 (**d**) cells transfecting with FHIT KO and FHIT HDR plasmids. The selected cell clones showed RFP. **e** DNA sequencing of the PCR amplified, sgRNA target site within the FHIT gene to verify FHIT KO. Two base deletions in HCC827 *FHIT*$^{-/-}$ and HDR insertion in H1650 *FHIT*$^{-/-}$ cells were observed. Both mutations caused frameshift null mutation in FHIT. **f** qPCR amplification to verify the FHIT overexpression in H460 *FHIT*^{OE} and H1299 *FHIT*^{OE} cells. GAPDH is the internal control.

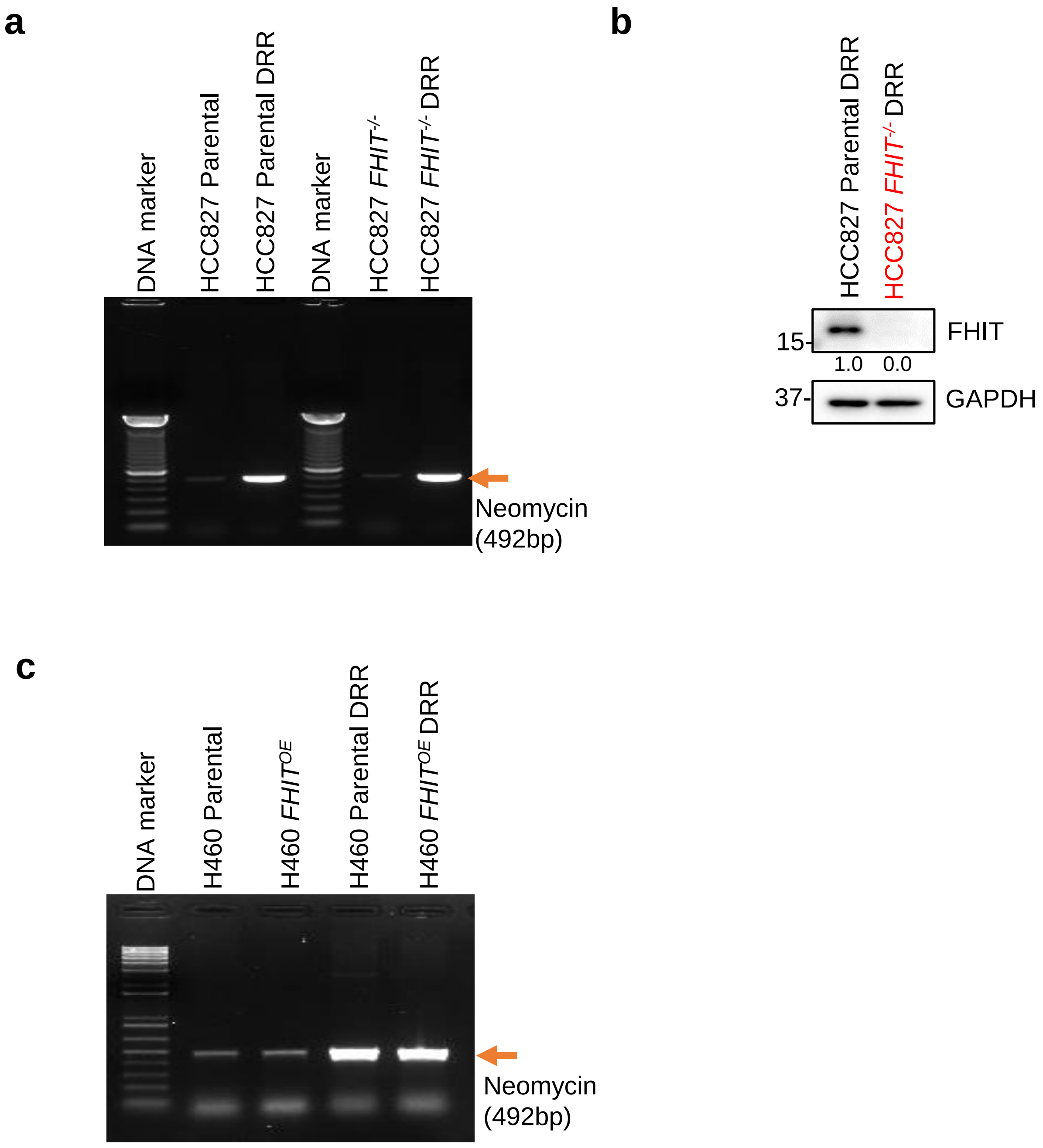

Supplementary Fig. 2. Generation of DRR cells. **a** PCR amplification of neomycin to verify the HCC827 FHIT-isogenic DRR cells. **b** Western blot to verify the FHIT KO in HCC827 FHIT-isogenic DRR cells. **c** PCR amplification of neomycin to verify the H460 DRR cells.

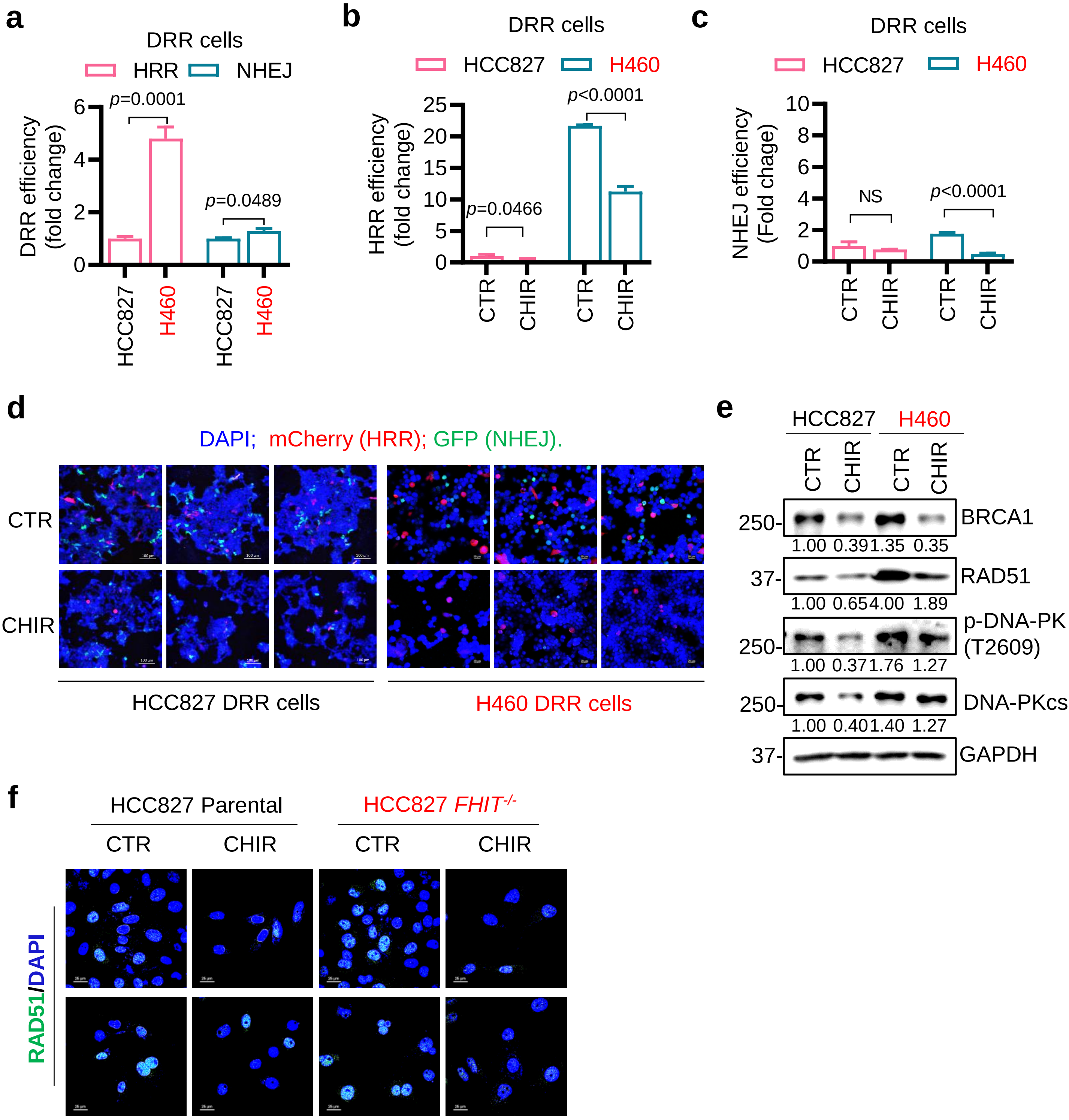

Supplementary Fig. 3. The inhibitory effect of GSK3βi on DRR. a-c Flow cytometry to analyze the DRR efficiency in HCC827 and H1460 DRR cells treated without (**a**) or with (**b**, **c**) 20 μM CHIR99021 for 24 h. The difference between the two groups was determined by unpaired two-tailed Student's t-test. NS denotes not significant. **d** The representative cell fluorescent images for (**b**, **c**), scar bar = 100 μm or 20 μm. The nuclear were stained Hochest33342. **e** Western blot to test the expression of the critical factors functioning in the HRR and NHEJ signaling (BRCA1, RAD51, P-DNA-PK (T2609) and DNA-PKcs) in HCC827 and H460 after treating with 20 μM CHIR99021 for 24 h. **f** Representative images of RAD51 Immunofluorescence staining in HCC827 FHIT-isogenic cells after treating with20 μM CHIR99021 for 24 h. GAPDH is the internal control.

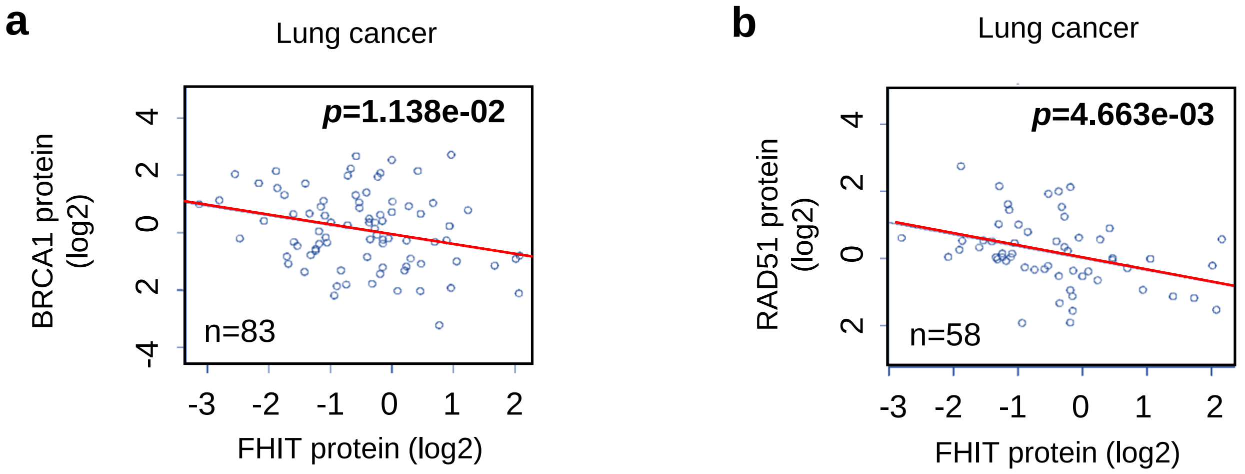

Supplementary Fig. 4. The clinical correlation of FHIT with BRCA1 and RAD51. a The relationship between FHIT protein and BRCA1 protein in lung cancer patients was obtained from the Linked Omics based on the TCGA data. **b** The relationship between FHIT protein and RAD51 protein in lung cancer patients was obtained from the Linked Omics based on the TCGA data.

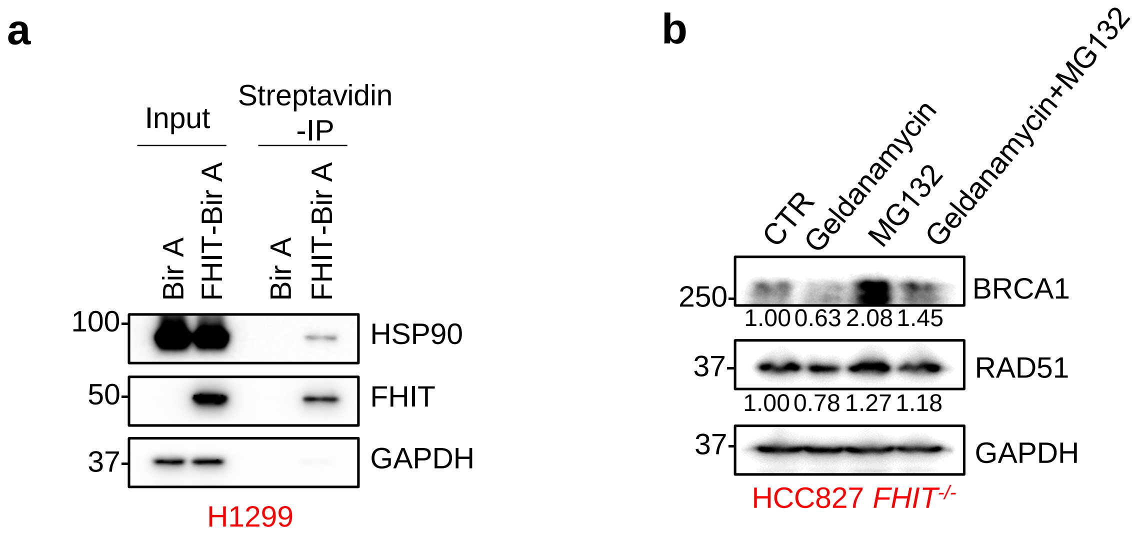

Supplementary Fig. 5. HSP90 and the regulation of BRCA1 and RAD51 protein stabilities. a Using Bio-ID to detect the interaction between FHIT and HSP90 in H1299 cells. **b** Western blot to detect the protein level of BRCA1 and RAD51 in HCC827 *FHIT*$^{-/-}$ cells treated with 125 nM geldanamycin for 24 h, 10 μM MG132 for 8 h or combine them.

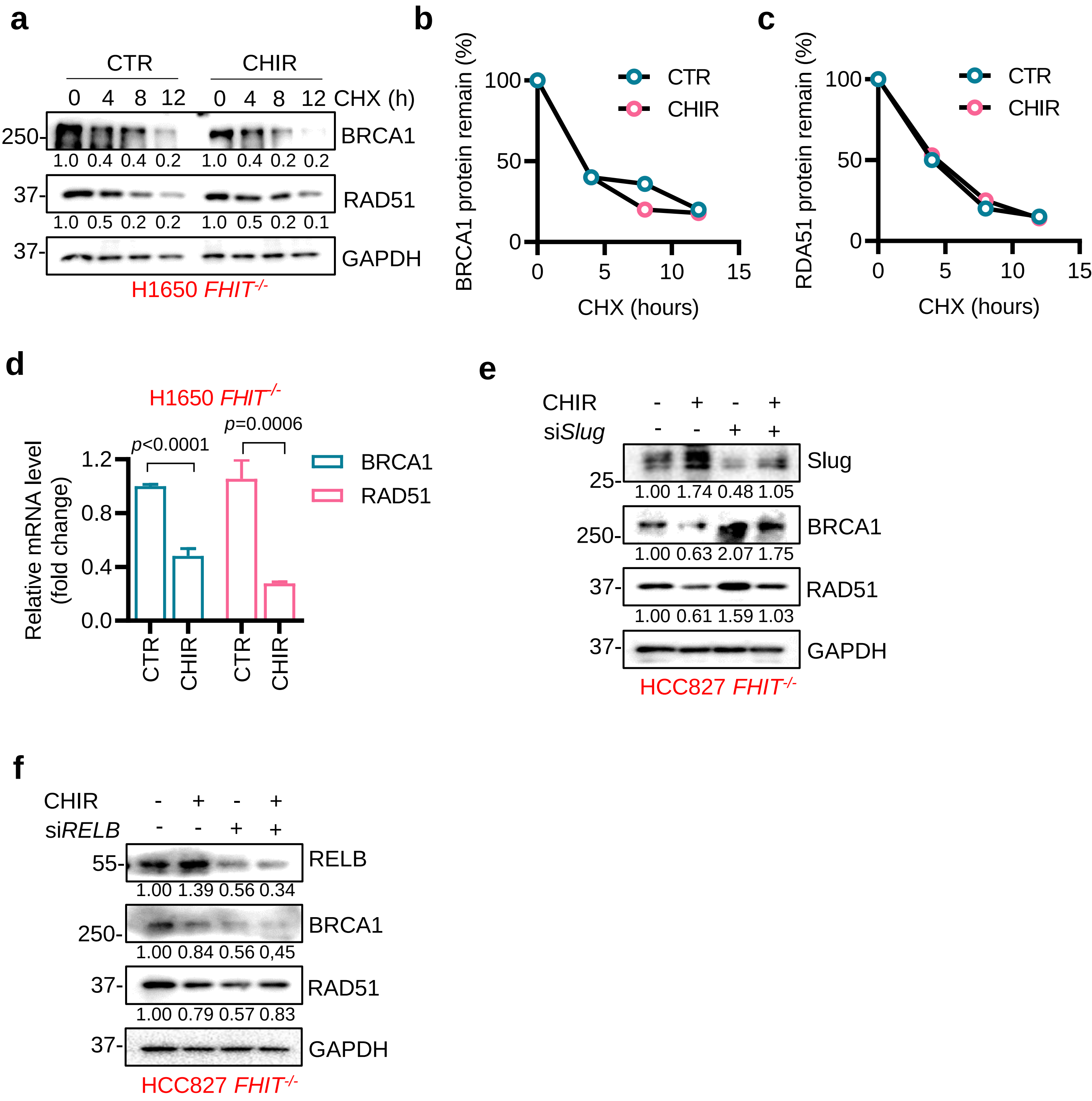

Supplementary Fig. 6. Regulation of GSK3β inhibitor on the BRCA1 and RAD51. **a-c** Effect of GSK3βi on the half life of BRCA1/RAD51. After H1650 *FHIT*$^{-/-}$ cells treated with or without 20 μM CHIR99021 30 min-post 50 μg/mL CHX incubated for the indicated times, Western blot to determine the protein levels of BRCA1 (**a**) and Image J software to quantitate the percentage of BRCA1 and RAD51 protein remain (**b, c**). **d** qPCR to analyze the mRNA level of BRCA1 and RAD51 after H1650 *FHIT*$^{-/-}$ treated with 20 μM CHIR99021 for 24 h. The difference between the two groups was determined by unpaired two-tailed Student's t-test. **e-f** Western blot to analyze the effect of si*Slug* (**e**) and si*RELB* (**f**) on CHIR99021-induced BRCA1 and RAD51 down-regulation. GAPDH is the internal control.

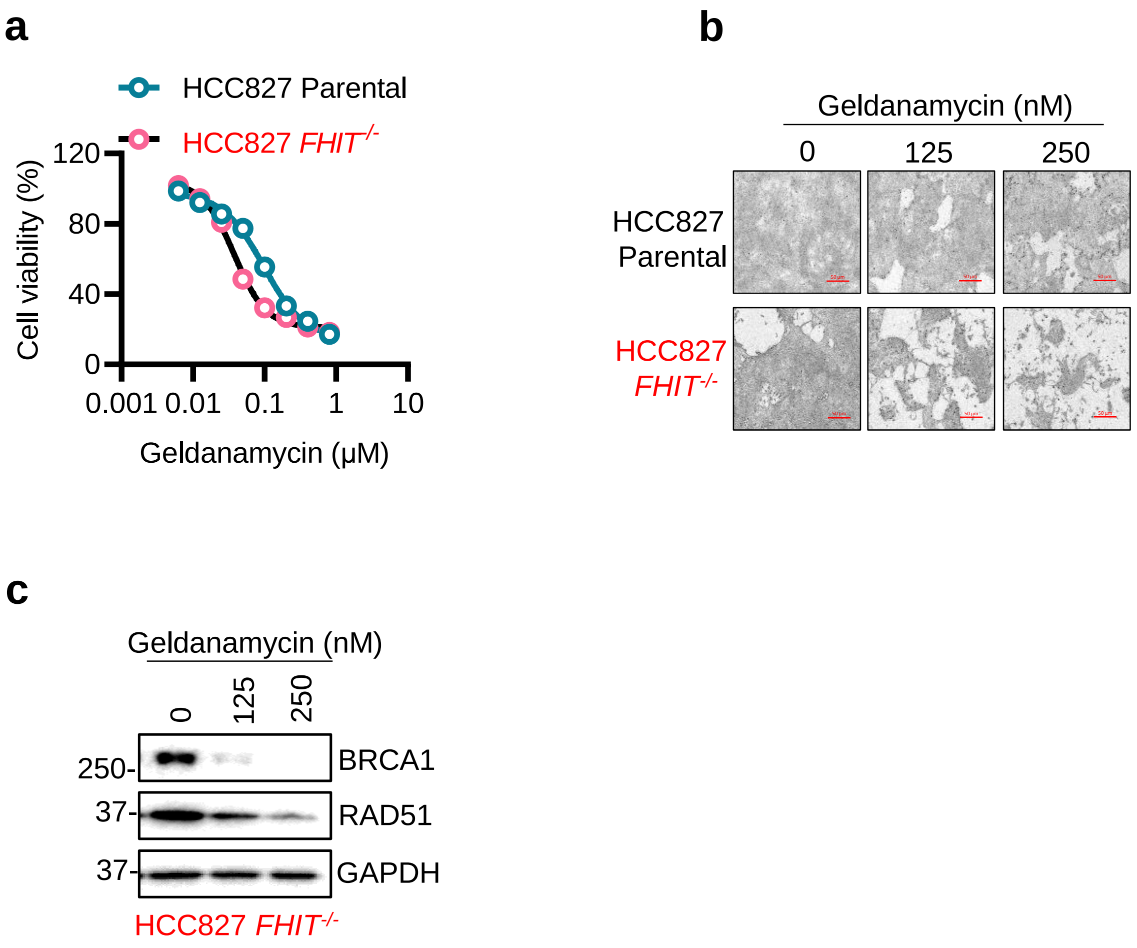

Supplementary Fig. 7. Involvement of DSB repair in the synthetic lethality between FHIT and HSP90. **a** The cell viability of HCC827-FHIT isogenic cells after treating with various concentrations of Geldanamycin for three days was detected by Alarma blue assay. **b** The representative images of HCC827 FHIT-isogenic cells after treatment with 125 nM Geldanamycin and 250 nM Geldanamycin for 48 h, scar bar = 50 μm. **c** Western blot to analyze the protein levels of BRCA1 and RAD51 in HCC827 *FHIT*$^{-/-}$ cells treated with 125 nM or 250 nM Geldanamycin for 48 h. GAPDH is the internal control.

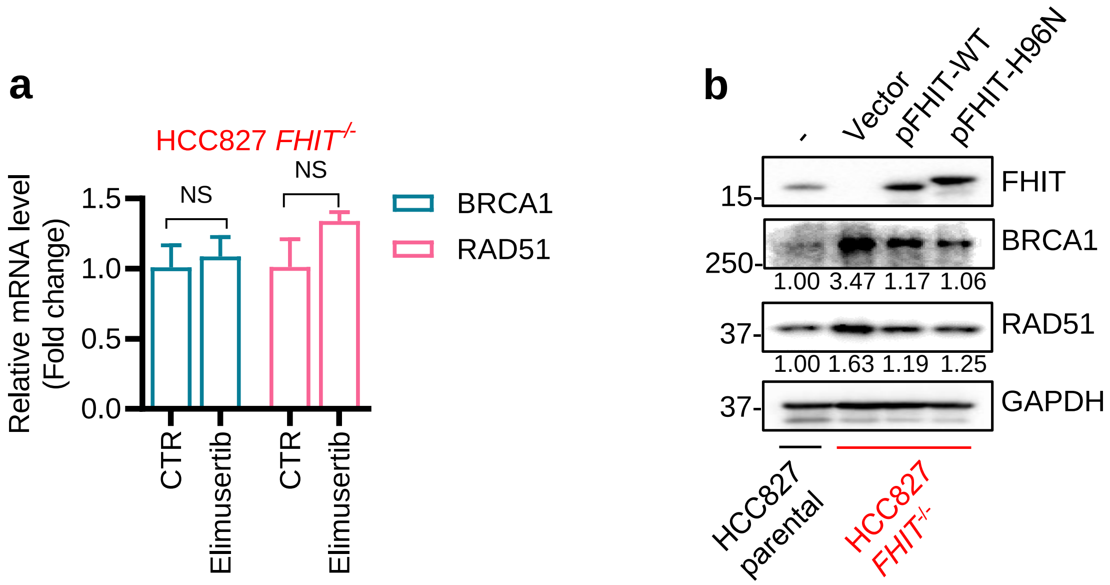

Supplementary Fig. 8. Analysis ATR inhibitor effect on HRR gene mRNA level and effect of FHIT hydrolase activity on HRR protein stability. a The HCC827 *FHIT*$^{-/-}$ cells were treated with 0.2 μM Elimuseertib for 12 hours and tested the BRAC1, RAD51 mRNA by qPCR. NS denotes Not Significant. **b** Western blot to detect the protein level of BRCA1 and RAD51 after the HCC827 *FHIT*$^{-/-}$ cell transfected with pFHIT-WT or pFHIT-H96N mutant plasmid for 48 h. GAPDH is the internal control.

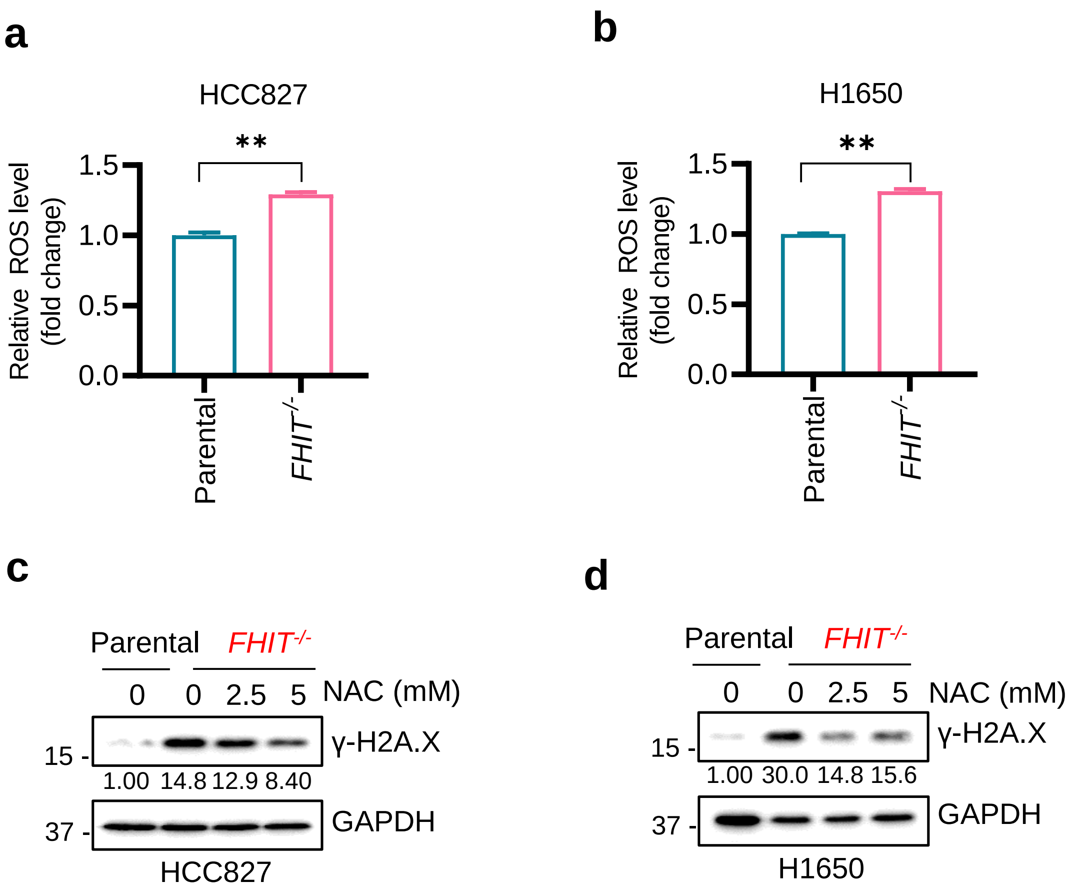

Supplementary Fig. 9. The effect of ROS on DNA damage in *FHIT*$^{-/-}$ lung cancer cells. a, b The ROS levels of HCC827 and H1650 FHIT-isogenic cells were detected by Flow Cytometry. ∗∗*P*<0.05. **c, d** Western blot to analyze the protein levels of γ-H2A in HCC827 *FHIT*$^{-/-}$ cells treated with N-acetylcysteine (NAC) for 48 h. GAPDH is the internal control.

Supplementary Table 1. Information of antibodies used in this study

Antibodies	Suppliers	Cat. No	Dilution for WB	Dilution for IF
FHIT	IBL International	JP18163	10 µg/mL	/
HA	Cell Signaling Technology	3724s	1:1000	/
GSK3α	Beyotime	AG2065	1:1000	/
GSK3β	Cell Signaling Technology	9832s	1:1000	/
P-GSK3α/β (Y216/Y279)	Beyotime	AF1522	1:1000	/
Cleaved Caspase-3	Cell Signaling Technology	9664s	1:500	/
PARP	Cell Signaling Technology	9532s	1:1000	/
γ-H2A.X	Cell Signaling Technology	9718s	1:1000	1:250
RAD51	Invitrogen	PA5-27195	1:1000	1:250
BRCA1	Santa Cruz Biotechnology	Sc-6954	1:200	/
P-BRCA (S423)	Beyotime	AB600	1:500	/
HSP90	Thermofisher	37-9400	1:1000	
DNA PKcs	Santa Cruz Biotechnology	Sc-5282	1:1000	/
P-DNA PK (Thr2609)	Invitrogen	PA1	1:1000	/
ATR	Cell Signaling Technology	13934s	1:1000	/
P-ATR (S428)	Cell Signaling Technology	2853s	1:1000	/
GAPDH	Santa Cruz Biotechnology	sc-365062	1:2000	/
Snail	Cell Signaling Technology	3879	1:1000	/
Slug	Cell Signaling Technology	9585	1:1000	/
RELB	Cell Signaling Technology	4922s	1:2500	/
Goat anti-mouse IgG, (H+L), peroxidase Conjugated	Thermo Scientific	31460	1:5000	/
Goat anti-rabbit IgG, (H+L), peroxidase Conjugated	Thermo Scientific	31430	1:5000	/
Alexa Fluor 488 donkey anti-rabbit IgG (H+L)	Invitrogen	A21206	/	1:1000

Supplementary Table 2. The sequence information of the PCR primers used in this study

Target	Primer sequence
FHIT-Forward	5’-CTGGAGTTCAGTGGATGGTAAA-3’
FHIT-Reverse	5’-CCGGGATATGAAAGGGAAGAAA-3’

Supplementary Table 3. The sequence information of the siRNAs used in this study

Target	siRNA sequence
FHIT	Sense-5'-rArUrUrCrCrArGrCrArArArArGrArGrCrUrArUrUrGrCrCAA-3’<br>Antisense-5'-rUrUrGrGrCrArArUrArGrCrUrCrUrUrUrUrGrCrUrGrGrArArUrUrC-3'
GSK3α	Sense-5'-rGrCrGrArGrArArGrArArArGrArCrGrArGrCrUrUrUrArCCT-3’<br>Antisense-5'-rArGrGrUrArArArGrCrUrCrGrUrCrUrUrUrCrUrUrCrUrCrGrCrCrA-3'
GSK3β	Sense-5'-rGrArUrArCrGrUrCrArGrUrGrGrUrCrUrArArArArUrGrCTA-3’<br>Antisense-5'-rUrArGrCrArUrUrUrUrArGrArCrCrArCrUrGrArCrGrUrArUrCrArA-3'

Supplementary Table 4. The sequence information of the RT-qPCR primers used in this study

Targets	Primer sequence
GAPDH-qPCR Forward	5'-GTGGACCTGACCTGCCGTCT-3'
GAPDH-qPCR Reverse	5'-GGAGGAGTGGGTGTCGCTGT-3'
FHIT-qPCR Forward	5'-AACTGTCCTTCGCTCTTGTG-3'
FHIT-qPCR Reverse	5'-GTCTGAAACAAATCGGCCAC-3'
RAD51-qPCR Forward	5'-CAACCCATTTCACGGTTAGAGC-3'
RAD51-qPCR Reverse	5'-TTCTTTGGCGCATAGGCAACA-3'
BRCA1-qPCR Forward	5'-ACCTTGGAACTGTGAGAACTCT-3'
BRCA1-qPCR Reverse	5'-TCTTGATCTCCCACACTGCAATA-3'
